# Next generation sequencing reveals changes of the γδ T cell receptor repertoires in patients with pulmonary tuberculosis

**DOI:** 10.1038/s41598-018-22061-x

**Published:** 2018-03-02

**Authors:** Chaofei Cheng, Bei Wang, Lei Gao, Jianmin Liu, Xinchun Chen, He Huang, Zhendong Zhao

**Affiliations:** 1 MOH Key Laboratory of Systems Biology of Pathogens, Institute of Pathogen Biology, and Centre for Tuberculosis Research, Chinese Academy of Medical Sciences & Peking Union Medical College, Beijing, 100730 China; 2 Clinical Immunology Center, Chinese Academy of Medical Sciences & Peking Union Medical College, Beijing, 100730 China; 3 CAMS-Oxford University International Center for Translational Immunology, Chinese Academy of Medical Sciences & Peking Union Medical College, Beijing, 100730 China; 4 The Sixth People’s Hospital of Zhengzhou, Zhengzhou, 450015 China; 5 Department of Pathogen Biology, School of Medicine, Shenzhen University, Shenzhen, 518002 China

## Abstract

Tuberculosis (TB) is a severe global threat to human health. The immune protection initiated by γδ T cells play an important role in mycobacterial infection. Vaccines for *Mycobacterium tuberculosis (Mtb*) based on γδ T cells provide a novel approach for TB control. In our previous studies, we found a preponderant complementarity-determining region 3 (CDR3) sequence of the γδ T cell receptor (TCR) in TB patients, and successfully identified a tuberculosis antigen that can effectively activate γδ T cells with a reverse genetic strategy. However, due to the throughput limitation of the method we used, the information we obtained about the γδ TCR repertoire and preponderant CDR3 sequences was limited. In this study, we introduced next generation sequencing (NGS) to study the γδ TCR CDR3 repertoires in TB patients. We found that the CDR3δ tended to be more polyclonal and CDR3γ tended to be longer in TB patients; the γδ T cells expressing CDR3 sequences using a Vγ9-JγP rearrangement expanded significantly during *Mtb* infection. We also identified new preponderant CDR3 sequences during *Mtb* infection. This study comprehensively characterized the γδ T cell receptor repertoire changes, and provides useful information for the development of new vaccines and adjuvants against TB.

## Introduction

Tuberculosis (TB) caused by *Mycobacterium tuberculosis* (*Mtb*), the top infectious disease killer worldwide, remains a severe global threat to human health. In 2015, it was estimated that there were 10.4 million new TB cases and 1.4 million deaths due to the disease^1^. The control of TB lacks new diagnostics, new drugs, and new vaccines; of these, effective vaccines are of vital importance to the prevention of TB. Currently, the only vaccine that is available and widely used against TB is bacille Calmette–Guérin (BCG), which can effectively protect children against active TB diseases, such as TB meningitis, but its efficacy in protecting adults and the aging population against pulmonary tuberculosis is highly variable^2,3^. Therefore, safer and more effective vaccines against TB are urgently needed.

To develop efficient TB vaccines, the mechanism of immune protection against TB must be identified. Unconventional T cells, such as lipid-specific CD1-restricted T cells, MAIT cells, and γδ T cells, function at the early stage of *Mtb* infection^4^. Among the unconventional T cells, the γδ T cells are thought to play a critical role in anti-mycobacterial immunity^5,6^. The role of γδ T cells in the immune response to Mycobacterium tuberculosis was first reported in 1989^7^. More and more research has demonstrated that the activation of γδ T cells appear rapidly following *Mtb* infection, and γδ T cells provide protective immunity against *Mtb* infection with the combined properties of both innate and adaptive immunity^8–10^. Vaccines for *Mtb* based on γδ T cells provide a novel approach to TB control^11–13^.

Originally, phosphoantigens were reported to activate γδ T cells, and were considered to be the main γδ T cell receptor (TCR)-recognized antigens^14^. However, only a subset of the phosphoantigen-responsive γδ T cells mediate protective tuberculosis immunity^15^. Based on this research, we established a reverse genetics strategy and successfully identified BCG and *Mtb*-derived protein antigens, which were not only recognized by γδ TCR, but could also activate γδ T cells and exhibit cytolytic effector function against BCG-infected cells^16–18^. In this strategy, we used PCR and Sanger sequencing to find preponderant complementarity-determining region 3 (CDR3) sequences in pulmonary tuberculosis patients. However, due to the limitation of the throughput of this method, we only analyzed 10^2^–10^3^ sequences and obtained limited information about γδ TCR^19^.

Since the TCR repertoire is a mirror of the human immune response, its characteristics have been widely investigated in infectious and other diseases to study the state of the immune system and the progression of these diseases^20–23^. The γδ TCR repertoire in pulmonary tuberculosis and tuberculosis meningitis has been studied with flow cytometry and PCR^24,25^; however, with the low throughput of these methods^19^, the information obtained from these studies is also limited. With the development of next generation sequencing (NGS), the sequencing depth and read length increased tremendously. The introduction of NGS to the reverse genetics strategy of our study will make the analysis of the γδ T cell receptor repertoire more comprehensive and robust.

In this study, to investigate the profile of the γδ T cells repertoire and to identify more preponderant CDR3 sequences in tuberculosis patients, we analyzed the expressed T cell receptor γ chain (TRG) and T cell receptor δ chain (TRD) repertoire of circulating γδ T cells that were isolated from people with latent tuberculosis infection (LTBI) and active pulmonary tuberculosis (TB). Compared with the healthy controls (HC), we found some changes in the TRG and TRD repertoire of the TB patients: the CDR3δ tended to be more polyclonal; the length of CDR3γ had a trend to be longer; the frequency of TRGV9 and TRGJP usage increased; and the γδ T cells expressing CDR3 sequences using a Vγ9-JγP rearrangement expanded significantly during *Mtb* infection. Additionally, we found some new preponderant γδ TCR CDR3 sequences in the LTBI group, the TB group, and the LTBI&TB group, which are expected to be more representative than the preponderant CDR3 sequences we obtained in our previous studies and could be used to screen novel *Mtb-*derived protein ligands of γδ TCR. Taken together, this study comprehensively characterized the TRG and TRD repertoire of TB patients, and provides some information about the preponderant CDR3 sequences of specifically recognized *Mtb*-derived antigens, which may help to develop new vaccines and adjuvants against TB.

## Results

### Summary of the subjects and sequencing

**Figure 1:**
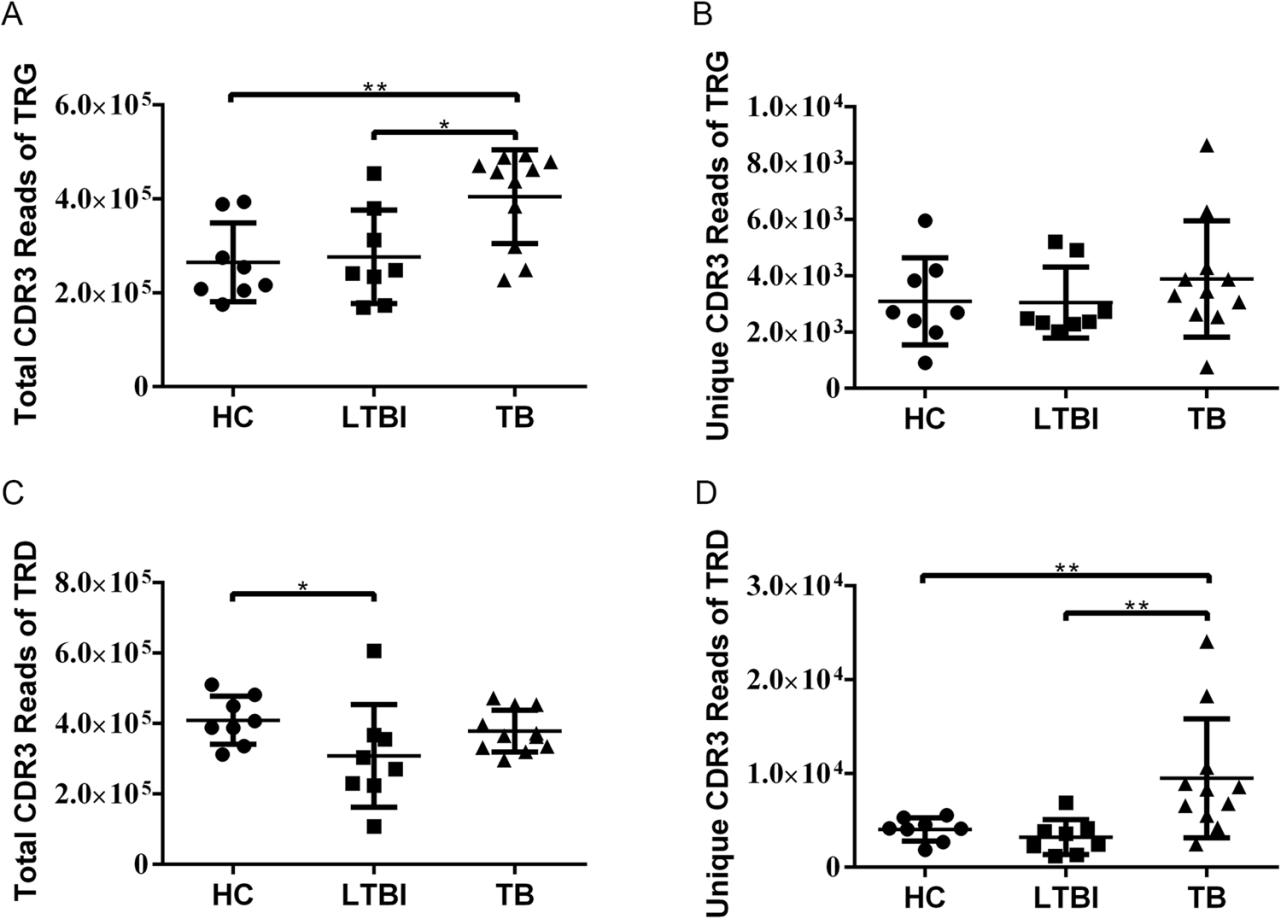
Overview of the CDR3 counts of the subjects. (**A**) The number of total CDR3γ sequences obtained from each of the samples in different groups. The bars show mean ± SD. Mann–Whitney test: *0.01 ≤ *p* < 0.05, **p < 0.01. The same as for (**A**) but showing the unique CDR3γ clonotypes (**B**), total CDR3δ sequences (**C**), and unique CDR3δ clonotypes (**D**) observed within each set.

To investigate the characteristics of the γδ T cell receptor repertoire of the three groups (HC, LTBI, and TB), 10 HC subjects, 8 LTBI subjects, and 12 TB patients were recruited for the study. The CDR3 of the γδ TCR underwent high-throughput sequencing with the Illumina MiSeq platform by iRepertoire^®^. Data from three samples (two HC subjects and one TB subject) were excluded because their reads were unusually fewer than the others due to an error in library preparation. The general information and clinical characteristics of the subjects are summarized in Table 1. Detailed information about the sequencing data of each sample is shown in Supplementary Table S1. The detailed information about mean sequencing depth, average CDR3 sequences, and average unique CDR3 reads for TRG and TRD in the three groups is shown in Supplementary Table S2. From the results, we found that the total CDR3 reads of TRG in TB was significantly more than that in HC and LTBI (Supplementary Table S2, Fig. 1A), while the number of unique CDR3 reads of TRG was similar among the different groups (Supplementary Table S2, Fig. 1B), indicating that clonal expansion of the γδ T cells expressing some specific CDR3γ sequences happened in TB patients, which may correlate with the *Mtb* infection. The differences of total CDR3 reads of TRD between the different groups were minor (Supplementary Table S2, Fig. 1C), while the unique CDR3 reads of TRD in TB was much more than that in HC and LTBI (Supplementary Table S2, Fig. 1D), suggesting that an increase in CDR3δ clonotypes in TB patients.

**Table 1:** General information of participants.

Study groups	Case No.	Sex (M/F)	Age range (mean, years)	Diagnostic criteria		
				Radiological images	AFB/sputum culture	ELISpot
Healthy control (HC)	8	3/5	25–46 (30.8)	Negative	—	Negative
Latent tuberculosis infection(LTBI)	8	2/6	24–55 (45)	Negative	—	Positive
Active Tuberculosis (TB)	11	8/3	19–58 (28.8)	Positive	Positive	—

### TRD CDR3 diversity increased in TB patients

**Figure 2:**
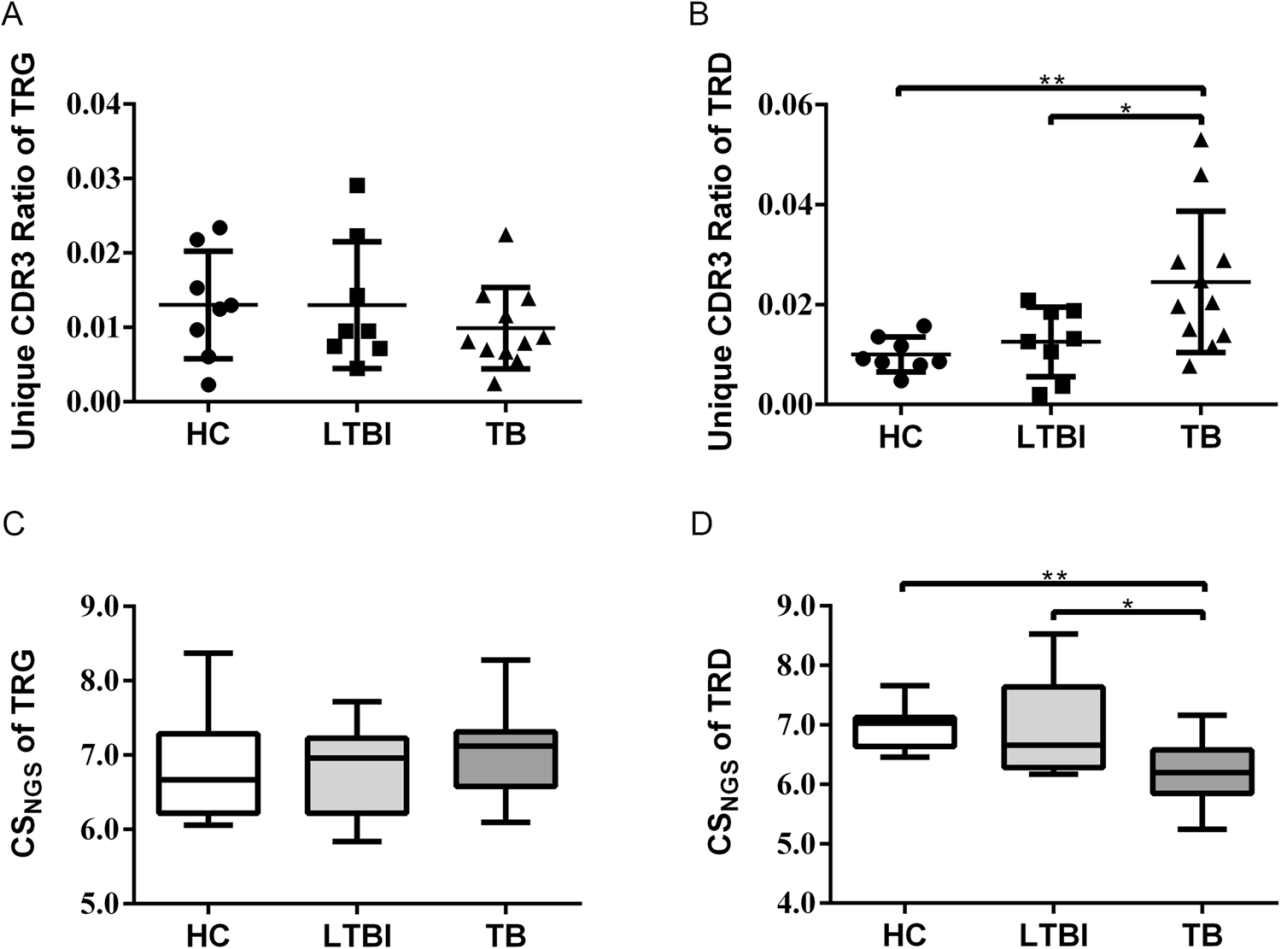
Diversity of the TRG and TRD repertoire in the different groups. (**A**–**B**) The unique CDR3 rations of the TRG **(A)** and TRD (**B**) repertoire in different groups were statistical analyzed. The unique CDR3 ration ranges from 0 to 1, with “0” representing the lowest diversity, while “1” represents the maximal diversity. The bars show mean ± SD. Mann–Whitney test: *0.01 ≤ *p* < 0.05, **p < 0.01. (**C–D**) Comparison of the next-generation-sequencing-spectratyping complexity scores (CS_NGS_) of TRG (**C**) and TRD (**D**) in different groups. Box plots present the results obtained by scoring the diversity of TRG and TRD in different groups (distribution and median values are shown).

The diversity of the TCR repertoire results from unique genetic diversification mechanisms and generates the potential of T cells to recognize millions of antigens. TCR repertoire diversity of γδ T cells can be affected by clonal expansion following the stimulation of *Mtb* antigens and affects a patient’s immune response to the pathogen. To estimate the repertoire diversity in the different groups, we took the unique CDR3 ratio as the indicator. Unique CDR3 ration was calculated by the number of unique TRG/TRD clones divided by the number of total sequences of each sample. Ideally, the unique CDR3 ration (also identified as the diversity index) ranges from 0 to 1, with “0” representing the lowest diversity, while “1” represents the maximal diversity. The results showed that there were no significant differences of TRG diversity between the three groups (Fig. 2A), while TRD diversity was significantly increased in the TB group when compared to the HC and LTBI groups (Fig. 2B). The TRD diversity of the LTBI group was also slightly increased when compared with the HCs, but the difference did not reach statistical significance (Fig. 2B). We also used the next-generation-sequencing-spectratyping complexity score (CS_NGS_) that was developed by Krell *et al*. to analyze the diversity of the TRG and TRD repertoires^26^, and the results were consistent with the unique CDR3 ratio (Fig. 2C,D). These data suggested that TRD diversity may positively correlate with the progression of tuberculosis.

### There is a trend of the length of CDR3γ to be longer in TB patients

**Figure 3:**
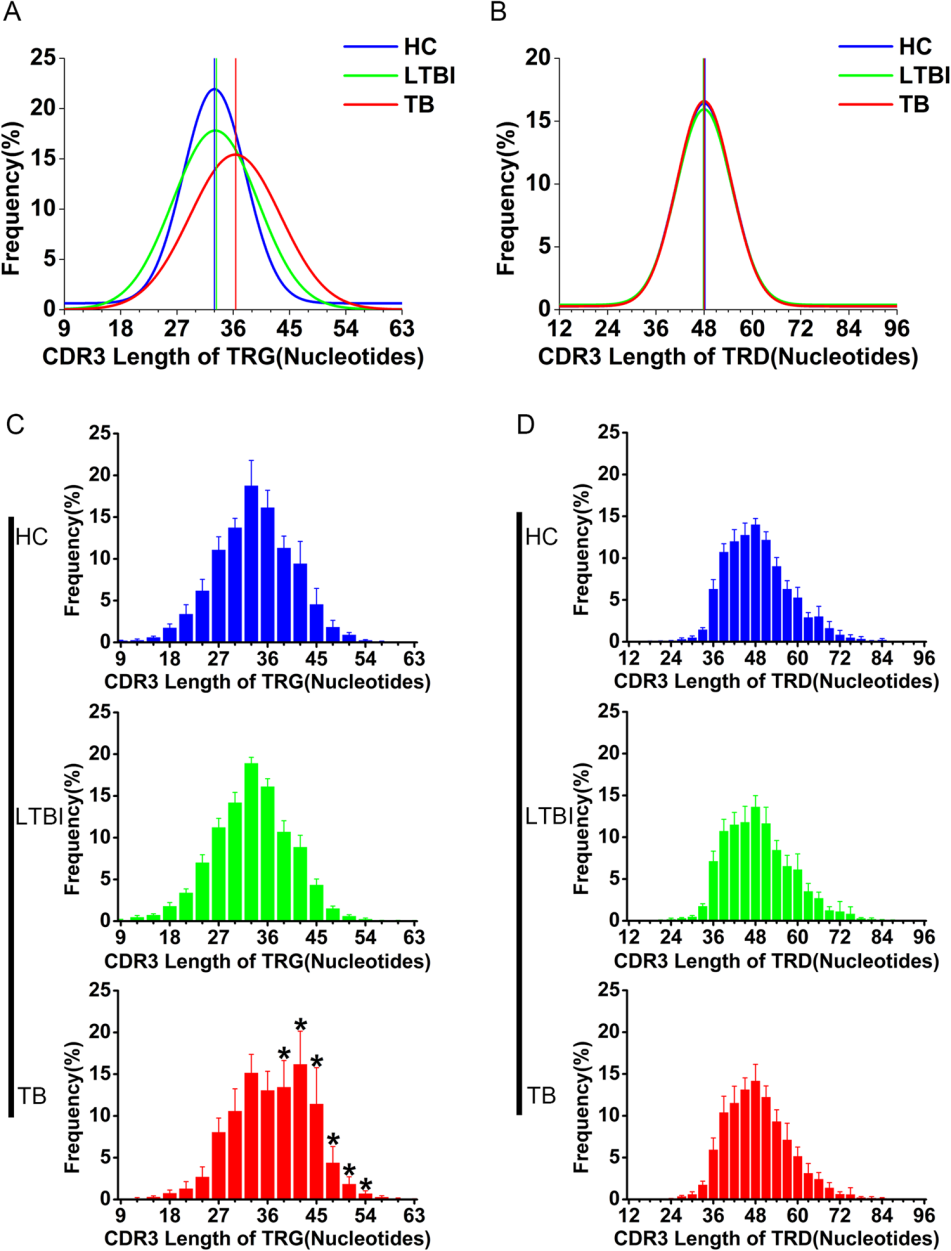
CDR3 length distribution of the TRG and TRD in the three groups. (**A**,**B**) The distribution patterns of unique CDR3γ length (**A**) and unique CDR3δ length (**B**) are displayed with line graphs. The lines represent the nonlinear curve fitting (Gauss function) to the frequency of TRG and TRD with different lengths in each group. The vertical line indicates the median of length in each group. The different colors represent the different groups. (**C**–**D**) The frequency of unique CDR3 sequences with different nucleotide lengths of TRG (**C**) and TRD (**D**) are shown with histograms. Bars show SD. The “*” above the length represents that the CDR3 sequences with this length have significant differences in the TB groups compared with that of the HC and LTBI groups (Mann–Whitney test: *0.01 ≤ *p* < 0.05, **p < 0.01).

The CDR3 regions of γδ TCR interact with antigens in a specific manner and are the most polymorphic region in these receptors. The enrichment of γδ T cells with a certain kind of CDR3 length is usually considered to be a result of the clonal expansion of immune-specific γδ T cells. To investigate the profiles of CDR3 lengths of the total CDR3 sequences in the γδ TCR repertoire of the three groups, we used polynomial fitting to assess the distribution patterns of the CDR3 length^27^. The distribution patterns showed that the length of the total CDR3γ sequences and the total CDR3δ sequences exhibited individual differences in different samples, and the length of the distribution patterns of the total CDR3γ sequences and the total CDR3δ sequences had no significant differences between different groups. We also used the Length Complexity Score (CS_L_) developed by Krell *et al*. to measure the CDR3 length polymorphisms of TRG and TRD. Since the types of the different CDR3 lengths that were observed in each of the TRGV and TRDV families are all more than 8, we did not set the maximum value of the numbers of the different CDR3 lengths contributing to the CS_L_. The CS_L_ scores of TRG (HC, 94 ± 9; LTBI, 95 ± 7; TB, 93 ± 9; mean ± standard deviation) and TRD (HC, 60 ± 4; LTBI, 58 ± 8; TB, 62 ± 6, mean ± standard deviation) have no significant differences between different groups. The distribution patterns of both unique CDR3γ sequence lengths and unique CDR3δ sequence lengths show an obvious Gaussian-liker distribution^28^. After nonlinear curve fitting (Gauss function), there was a trend of unique CDR3γ sequence lengths to be longer in the TB patients (Fig. 3A), and the frequency of unique CDR3γ sequences with 39, 42, 45, 48, 51, and 54 nucleotides increased significantly, especially the sequences with 42 nucleotides (Fig. 3C and Supplementary Figure 1). However, the distribution patterns of the unique CDR3δ lengths had no difference among different groups (Fig. 3B,D). Taken together, the above data suggested that long-term antigenic stimulation may lead to longer CDR3γ length and the clonotypes of CDR3γ with certain kinds of nucleotides length (39, 42, 45, 48, 51 and 54) undergo clonal expansion during *Mtb* infection. This result also implied that the γδ T cells expressing the longer CDR3γ sequences may play important roles in recognizing *Mtb*-specific antigens.

### The usage of gene segments changed in CDR3 of TRG/TRD in TB patients

**Figure 4:**
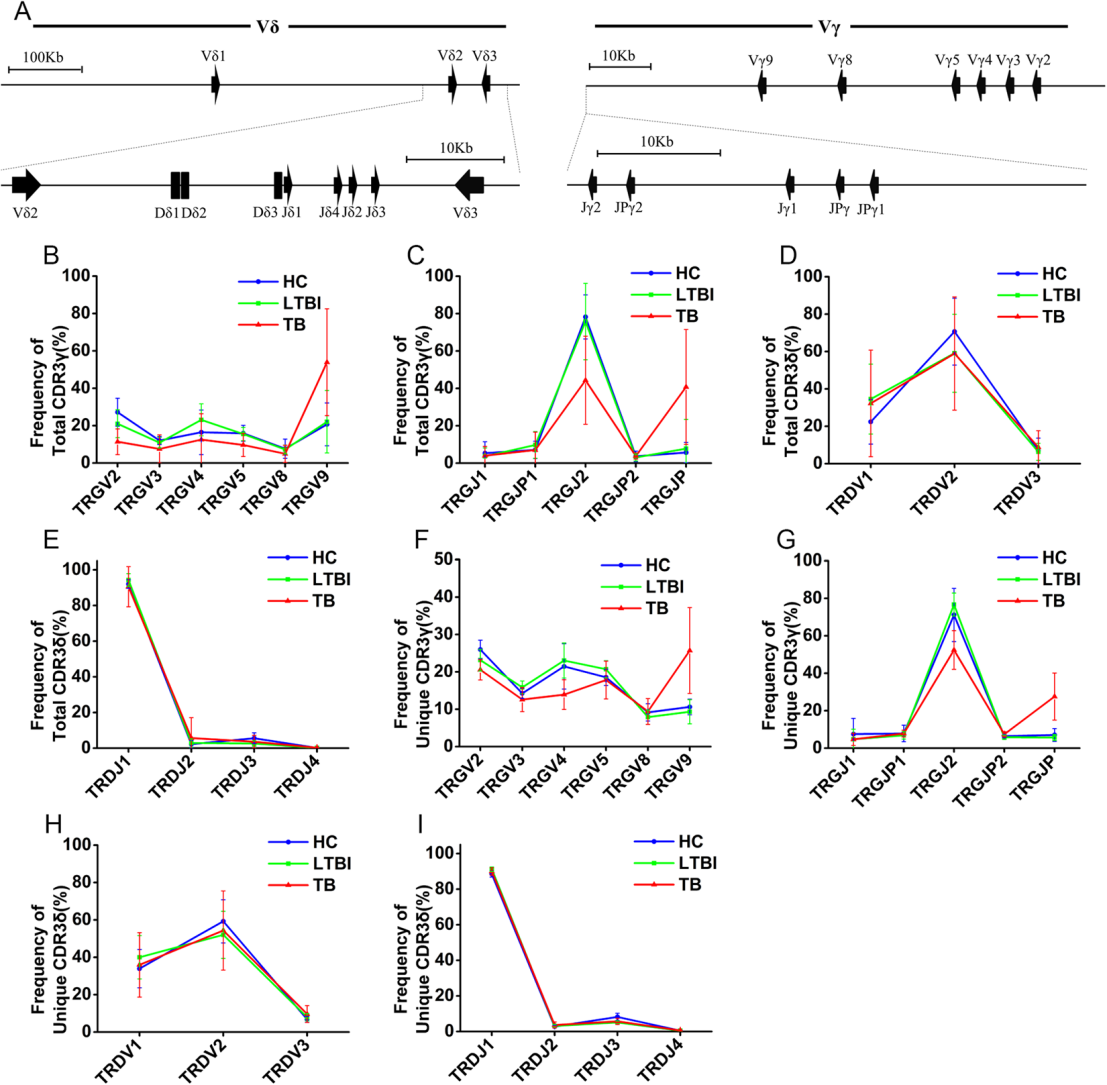
Individual V gene and J gene usage of TRG/TRD in different groups. (**A**) Genomic organization of the different gene segments comprising the human γδ TCR. (**B**–**E**) The frequency of usage of individual TRGV (**B**), TRGJ (**C**), TRDV (**D**), and TRDJ (**E**) genes in total CDR3 sequences are displayed. The lines indicate the average frequency of the different gene segment usage in each group and bars show SD. The different colors represent the different groups. (**F**–**I**) The same as for (**B**–**E**) but showing the frequency of usage of individual TRGV (**F**), TRGJ (**G**), TRDV (**H**), and TRDJ (**I**) genes in unique CDR3 sequences.

**Figure 5:**
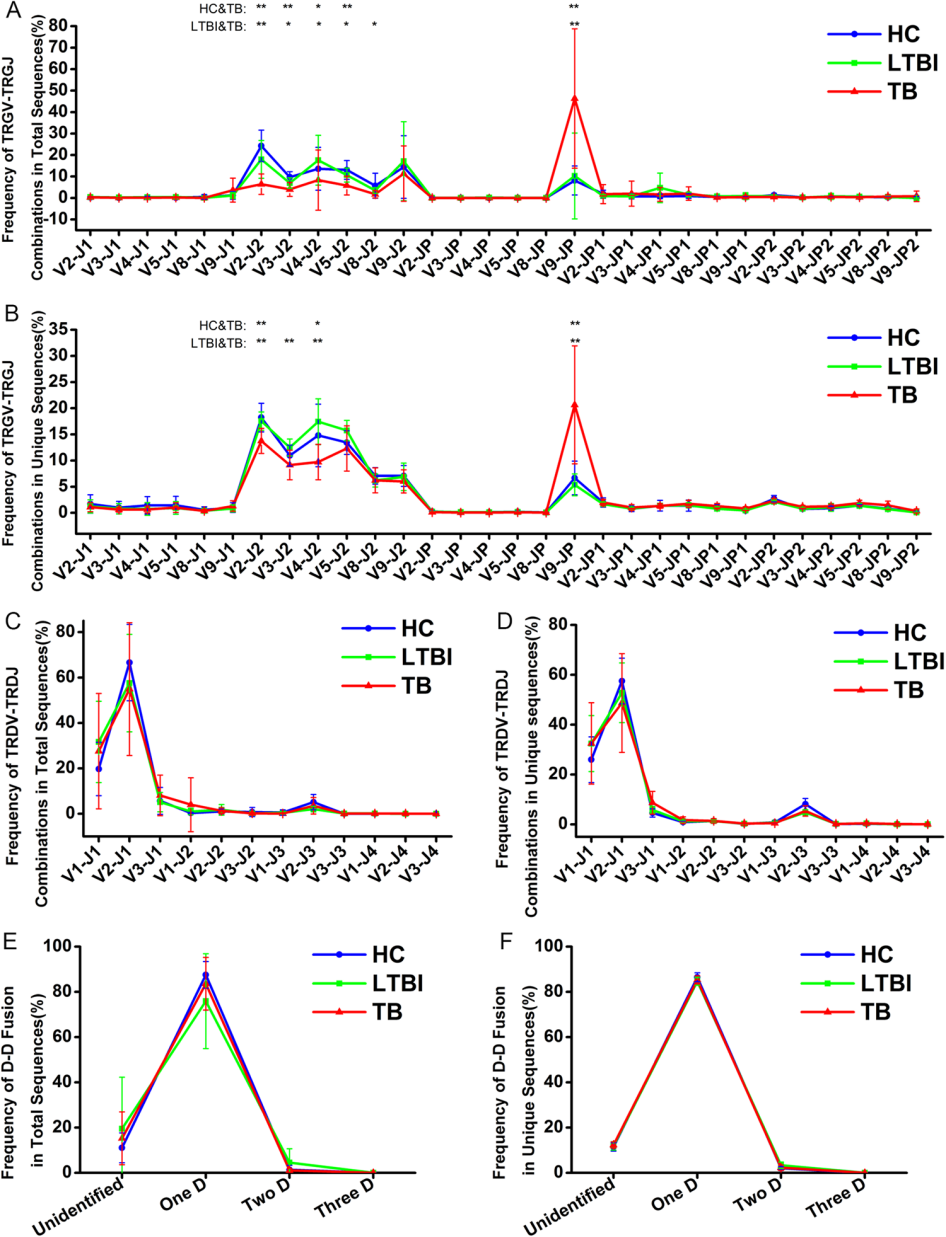
Different V-J combination of TRG/TRD and the D-D fusion pattern of TRD in different groups. (**A**,**B**) The frequency of CDR3γ sequences bearing different TRGV-TRGJ rearrangements in total CDR3 sequences (**A**) and unique CDR3 sequences (**B**) are displayed. The lines indicate the average frequency of the different V-J combinations in each group and the bars show SD. The “*” above the different TRGV-TRGJ rearrangements represents that the statistical analyses of this TRGV-TRGJ rearrangements between different groups (Mann–Whitney test: *0.01 ≤ p < 0.05, **p < 0.01). The different colors represent the different groups. (**C**,**D**) The same as for (**A**,**B**) but showing the frequency of CDR3δ sequences bearing different TRDV-TRDJ rearrangements in total CDR3 sequences (**C**) and unique CDR3 sequences (**D**). (**E**,**F**) The frequency of the D-D fusion pattern of the total TRD CDR3 sequences (**E**) and unique TRD CDR3 sequences (**F**) in all of the subjects are shown. The lines indicate the average frequency of the different D-D fusion in each group and the bars show SD. The different colors represent the different groups.

The combinatorial diversity of TRG and TRD arise from rearrangement of different gene segments present in the germline. Figure 4A illustrates the genomic organization of the V, D, and J gene segments of human TRG and TRD [reviewed in ref.^29^]. We assessed the usage of individual V genes and J genes of TRG and TRD in different groups. For total CDR3 sequences, the usage of the TRGV2, V3, and V5 segments slightly decreased, while the usage of the TRGV9 segment increased significantly in the TB group compared to the HC and LTBI groups; the usage of the TRGV4 and TRGV8 genes in the TB group also decreased in comparison to the LTBI group (Fig. 4B and Supplementary Figure 2); the usage of TRGJP increased significantly, while the usage of TRGJ2 decreased obviously in the TB group in comparison to the HC and LTBI groups (Fig. 4C and Supplementary Figure 3); the usage of different TRDV gene segments was similar between different groups (Fig. 4D and Supplementary Figure 4); and the usage of the TRDJ3 segment was decreased in the LTBI and TB groups when compared to the HC group (Fig. 4E and Supplementary Figure 5). For unique CDR3 sequences, the decreased usage of TRGV4 and the increased usage of TRGV9 in the TB patients was more obvious (Fig. 4F and Supplementary Figure 6); the decreased usage of TRGJ2 and the increased usage of TRGJP were also observed in the TB groups (Fig. 4G and Supplementary Figure 7); and the usage of TRDV and TRDJ segments was similar between the three groups, with the exception of the decreased usage of TRDJ3 in the TB and LTBI groups (Fig. 4H,I, Supplementary Figures 8 and 9). According to the above results, we proposed that with the stimulation of *Mtb*-derived antigens in the TB patients, the rearrangement of the TRGV and TRGJ genes may produce specific γδ T cells expressing certain TRGV and/or TRGJ gene segments. Considering the changes of TRGV9 and TRGJP usage in both the total sequences and the unique sequences in the TB group, we speculated that the γδ T cells expressing the TRGV9 and/or TRGJP gene segments were produced and expanded clonally with the stimulation of *Mtb*-derived antigens. To further confirm this interpretation, we analyzed the TRGV-TRGJ and TRDV-TRDJ combinations for the TRG and TRD repertoire in the three groups, respectively. We found that the CDR3γ sequences using TRGV9-TRGJP rearrangement increased significantly in the TB group for both the total CDR3γ sequences (Fig. 5A) and the unique CDR3γ sequences (Fig. 5B). The percentage of CDR3γ sequences using the TRGV9-TRGJP rearrangement was higher in the total CDR3γ sequences than that in the unique CDR3γ sequences in the TB group; this indicated that some γδ T cells with the CDR3γ sequences using the TRGV9-TRGJP rearrangement clonally expanded in the TB patients. The TRDV-TRDJ combination of the TRD repertoire was similar for both the total CDR3δ sequences (Fig. 5C) and the unique CDR3δ sequences (Fig. 5D) in the three groups. To further confirm this conclusion, we also used the combination complexity score (CS_C_) developed by Krell *et al*. to analyze the TRGV-TRGJ and TRDV-TRDJ combinations of the TRG and TRD repertoire in the three groups^26^. Since the number of reads is different in each sample, we used frequency instead of the reads to calculate the CS_C_. The results also showed that the CDR3γ sequences using TRGV9-TRGJP rearrangement increased significantly in the TB group (Supplementary Figure 10). Taken together, we drew the conclusion that the γδ T cells with the TCR using TRGV9-TRGJP rearrangement may play important roles in the immune response against *Mtb* infection. It has been reported that D gene segments are also very important for the potential diversity of TRD due to the rearrangement of different D gene segments and the three reading frames of each D gene segments. We analyzed the D-D fusion pattern of TRD in the three groups, but no obvious difference was observed in both the total CDR3 sequences (Fig. 5E) and the unique CDR3 sequences (Fig. 5F). This showed that the D-D fusion did not change with the antigens stimulation from *Mtb* in the TB patients, so the D-D fusion had no impact on the length distribution of the TRD repertoire.

### J trimming of TRG was shorter in TB patients

**Figure 6:**
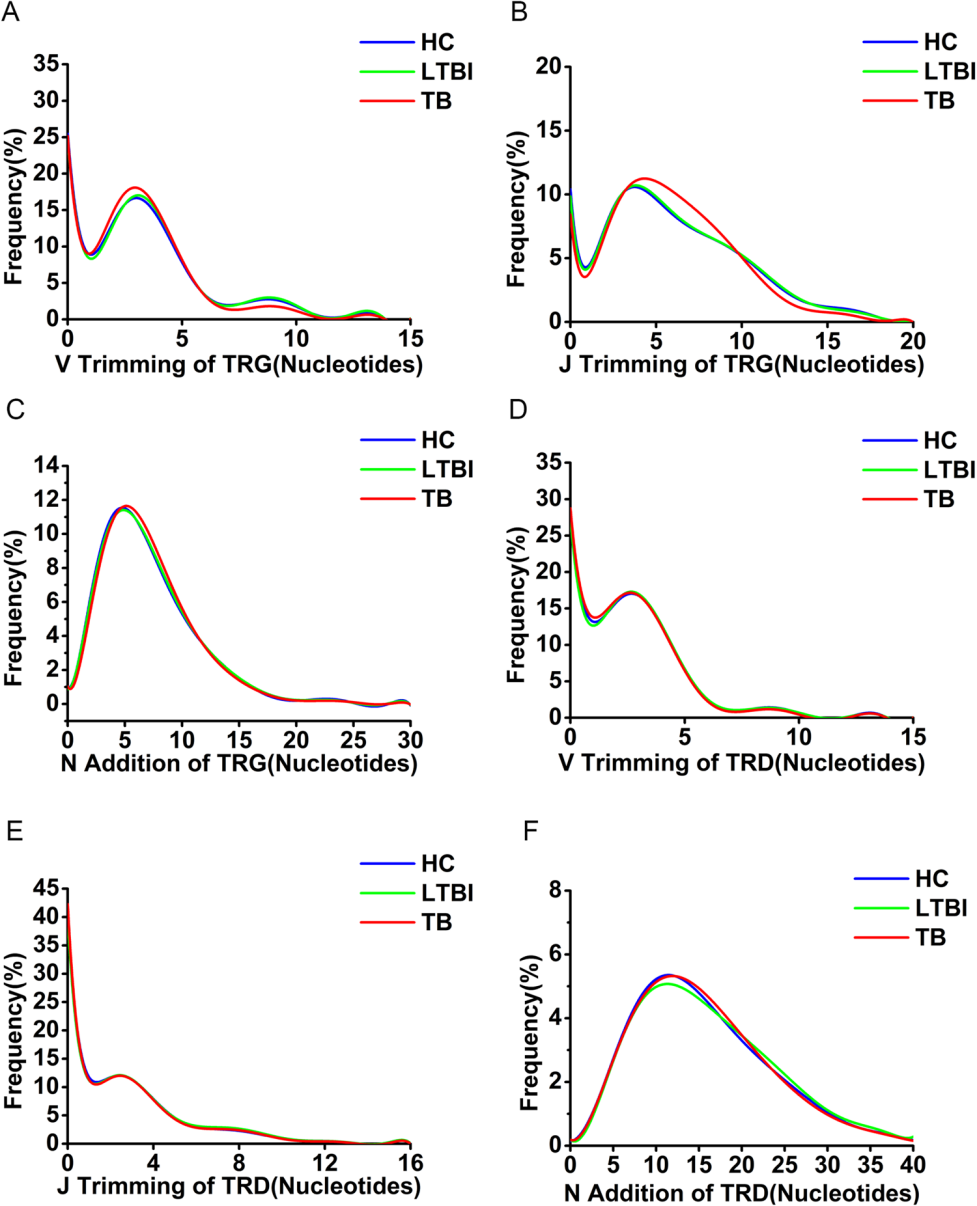
J trimming, V trimming, and N addition of the TRG and TRD in different groups. (**A**–**C**) V trimming (**A**), J trimming (**B**), and N addition (**C**) of TRG in the different groups are shown with the line graph. The smooth lines represent a linear polynomial fit (ninth order) to the data of the nucleotides length of J trimming, V trimming, and N addition in each group. The different colors represent the different groups. (**D**–**F**) The same as for (**A**–**C**) but showing the V trimming (**D**), J trimming (**E**), and N addition (**F**) of TRD in different groups.

Besides combinatorial diversity, the diversity of the TRG and TRD genes also arise from junctional diversity, which is caused by differential trimming of the termini in the recombined segments with an exonuclease and inserting template-independent nucleotides with terminal transferase (N addition). To estimate the junctional diversity of the TRG/TRD repertoire among the different groups, we analyzed the J trimming, V trimming, and N addition of TRG and TRD in the different groups. For total CDR3 sequences, there was no distinct regularity and no significant differences of J trimming, V trimming, and N addition between the three groups. For unique CDR3 sequences, there was no obvious differences in J trimming, V trimming, and N addition of TRD (Fig. 6D–F), and V trimming (Fig. 6A) and N addition of TRG (Fig. 6C). However, the nucleotides of TRG J trimming were slightly less in the TB group compared to the HC and LTBI groups (Fig. 6B). The shortened J trimming of TRG may be the reason for the increased length of TRG that we observed in the above results (Fig. 3A).

### Preponderant CDR3 sequences during *Mtb* infection

It has been reported that γδ T cells are present in increased proportions in the peripheral blood of a fraction of tuberculosis patients^30^ and some clonotypic γδ T cells exhibited clonal expansion during BCG infection and re-infection^31^. These studies suggested that clonotypic γδ T cells also clonally expanded during *Mtb* infection and these γδ T cells carried CDR3 sequences specifically recognized *Mtb*-derived antigens. These specific CDR3 sequences, which are also known as preponderant CDR3 sequences, may be critical for the recognition of *Mtb*-derived antigens and instructive for the development of new vaccines. To identify preponderant CDR3 sequences of the γδ TCR in *Mtb* infection, we analyzed the sequences obtained by NGS. To find preponderant CDR3 sequences in the LTBI, TB, and LTBI&TB groups, we chose unique CDR3 sequences to analyze so as to avoid bias caused by PCR amplification. We first identified the particular sequences in the LTBI, TB, and LTBI&TB groups, and then defined the sequences that occurred in over 50% of the samples as the preponderant CDR3 sequences. We summarize the preponderant CDR3 sequences in Table 2. There were 9, 15, and 13 preponderant CDR3γ sequences in the LTBI, TB, and LTBI&TB groups. However, the preponderant CDR3δ sequence was relatively less. There was no preponderant CDR3δ sequence found in the LTBI group, and there were only 3 in both the TB and LTBI&TB groups. The most frequently used preponderant CDR3γ sequences were ALWEVHALGKKIKV and ALWEVVRELGKKIKV; their frequency was up to 72.7%. We also found that the preponderant CDR3γ sequences in the TB group were all composed of 13aa, 14aa, and 15aa. This result was consistent with the length distribution analysis of TRG, in which the CDR3γ sequences with 39, 42, and 45 nucleotides also exhibited increased frequency (Fig. 3). The preponderant CDR3 sequences we identified may provide useful information for the development of new vaccines.

**Table 2:** Preponderant CDR3 sequences in Mtb infection.

Group	TRG CDR3 Amino acid sequences	Frequency	TRD CDR3 Amino acid sequences	Frequency
LTBI	ACYYKKL	50.0%	None	
	ALWEVQLKL	50.0%		
	ATWETGWFKI	50.0%		
	ATWDLHYYKKL	50.0%		
	ATWDYGYYKKL	50.0%		
	ALWRKL	50.0%		
	ATWDRRRL	50.0%		
	ATWDLPYYYKKL	50.0%		
	ATFDYKKL	50.0%		
TB	ALWEVHALGKKIKV	72.7%	ACDPLLGDNTDKLI	54.5%
	ALWEVVRELGKKIKV	72.7%	ACVLLGDKADKLI	54.5%
	ALWEVFGELGKKIKV	63.6%	ACDTATGGPSSWDTRQMF	54.5%
	ALWEARRELGKKIKV	63.6%		
	ALWEVHLELGKKIKV	54.5%		
	ALWEPKLGKKIKV	54.5%		
	ALWEVWQELGKKIKV	54.5%		
	ALWEGGLGKKIKV	54.5%		
	ALWEVSSELGKKIKV	54.5%		
	ALWEESELGKKIKV	54.5%		
	ALWVSELGKKIKV	54.5%		
	ALWEVVKLGKKIKV	54.5%		
	ALWEEGQELGKKIKV	54.5%		
	ALWEVISELGKKIKV	54.5%		
	ALWEVAGLGKKIKV	54.5%		
LTBI&TB	ALWEDGKL	63.2%	AFKVGYWGILTDKLI	57.9%
	ATSTPGWFKI	63.2%	ACDTLTGGSYTDKLI	57.9%
	ATWDDLKKL	57.9%	ANTGGYGTDKLI	52.6%
	AFPAGKL	57.9%		
	ALWEGLQELGKKIKV	57.9%		
	ALWEVQRQELGKKIKV	57.9%		
	ALWDSELGKKIKV	52.6%		
	ALWEVHSELGKKIKV	52.6%		
	ALWGIQELGKKIKV	52.6%		
	ATWDRLRYYKKL	52.6%		
	ATWDRPAYYKKL	52.6%		
	ATWDSQYYKKL	52.6%		
	ATWDRKYYKKL	52.6%		

## Discussion

It has been widely reported that γδ T cells, especially Vγ9Vδ2 T cells, play an important role in the protective immune responses to *Mtb* infection^24,32^. They can expand in response to the stimulation of *Mtb* antigens *in vitro* and *in vivo*^13,33,34^. In our previous work, we demonstrated γδ T cells’ response to Mycobacterium tuberculosis in pulmonary tuberculosis patients using preponderant complementary determinant region 3 sequences^16^. The γδ TCR repertoire in patients with pulmonary tuberculosis and tuberculosis meningitis has been studied with the flow cytometry and PCR^24,25^, but due to the natural limitation of these methods^19^, the information achieved from these studies is not enough to comprehensively illustrate the γδ TCR repertoire changes during *Mtb* infection. With the development of NGS, deep sequencing has already been applied in the analysis of TCR repertoire and immunoglobulin repertoire^35–37^. Li and colleagues have profiled the β TCR repertoire differences of PBMCs and pleural effusion mononuclear cells (PEMCs) from one of the TB patients using NGS^38^. To better characterize the changes of γδ TCR repertoire in TB infection, we investigated the γδ TCR repertoire in pulmonary tuberculosis patients by high-throughput sequencing using the Illumina MiSeq platform combined with ARM-PCR. With the combination of the semi-quantitative property of ARM-PCR and the high sequencing depth of NGS, we characterized the profile of the γδ T cells repertoire in TB patients at the level of sequence resolution for the first time.

Due to the scarcity of the sample resources, the recruits in our study were limited. We were not able to recruit subjects that were well-matched for age and sex in the beginning. In order to exclude the variation caused by age and sex distribution in the different groups, we analyzed the differences of age or sex among the different groups with analysis of variance and the Chi-square test, respectively. We found that there were no differences in the age and sex distributions among the HC, LITB, and TB groups, except that the age distribution in the LTBI group was different from that in the HC and TB groups with significance (Supplementary Tables 3 and 4). Since the conclusions we drew were mostly focused on the differences between the HC and TB groups, we think that the variables, such as age and sex, will not affect the conclusions in the manuscript.

From the sequences we obtained, the total number of CDR3γ sequences in the TB patients was more than that in the HC and LTBI groups, while the unique CDR3γ sequences were similar among the three groups. However, this quantitative discrepancy was not present in the total CDR3δ sequences and unique CDR3δ sequences, indicating that the clonal expansion of the γδ T cells expressing some specific CDR3γ sequences in the TB patients occurred, which may have a correlation with the *Mtb* infection. The higher percentage of CDR3γ sequences bearing the TRGV9-TRGJP rearrangement in the total CDR3γ sequences than that in the unique CDR3γ sequences also supported this conclusion. For CDR3δ sequences, the number of unique CDR3δ sequences was significantly increased in the TB group, suggesting increased TRD CDR3 diversity in this group, and this conclusion was validated when we used unique CDR3 ration to evaluate the diversity of the TRD repertoire; this may also explain the little preponderant CDR3δ sequences in the TB and LTBI&TB groups, but no preponderant CDR3δ sequence in the LTBI group. The increased TRD diversity may reflect that the TRD tend to be polyclonal under the stimulation of *Mtb*-derived antigen.

The CDR3 length, which is affected by the thymic selection, has a profound effect on the shape of TCR, and the analysis of CDR3 length variation might shed light on the structure-function relationships of different TCRs recognizing specific antigens^39^. The unique CDR3 length distribution of the γ chain and δ chain exhibit a single/multi-peak-shaped pattern (Gaussian distribution), just as the profile of the gene melting spectral pattern of the TCR beta variable gene family in PBMCs from healthy donors^28^. In our results, there was a trend of CDR3γ to be longer in the TB group, and CDR3γ sequences with a specific number of nucleotides were obviously increased in the TB group. This result suggested that the increased CDR3γ length may be correlated with the *Mtb* infection, and the γδ T cells expressing CDR3γ sequences with longer lengths may play important roles in the immune response against *Mtb* infection. The increased length of CDR3γ and the enrichment of CDR3γ sequences with 39, 42, and 45 nucleotides were also demonstrated by the preponderant CDR3 sequences we found in the TB group (Table 2), all of which are composed of 13, 14, and 15 amino acids. As for the mechanism by which TRG tended to be longer in the TB group, we thought that the fewer nucleotides of J trimming we observed in TRG (Fig. 6B) may be one reason.

When we analyzed the gene fragment usage of the CDR3γ and CRD3δ, we found skewed usage of TRGV9 and TRGJP in the TB patients for both total sequences and unique clonotypes. For CDR3δ, we did not observe obviously skewed usage of gene segments between the different groups, although it was thought to account more for the diversity of the γδ TCR repertoire and play critical roles in the formation of γδ TCR-antigen complexes^40–44^. The skewed usage of TRGV9 is consistent with previous studies that demonstrated that reactivity towards *Mtb* was an exclusive property of Vγ9-bearing γδ T cells that coexpress Vδ2^45^. The results suggested that the γδ T cells using specific TRGV9 and/or TRGJP gene segments were important for the recognition of *Mtb*-derived antigens and the γ chain may be more important in antigen recognition than the δ chain. Bukowski *et al*. had demonstrated that the TCR γ chain played a crucial role in antigen recognition by γδ TCR, at least in pyrophosphate antigen recognition^46^. Similarity, Van Rhijn *et al*. also demonstrated that antigenic selection had taken place for certain TCR γ chains, and that the TCR δ chain might be less important for specific antigen recognition during the stimulation of antigens when they studied the highly diverse TCR δ chain repertoire in bovine subjects^47^. According to the results of the V-J combination, we inferred that the γδ T cells expressing Vγ9-JγP rearrangement from peripheral blood recognized the *Mtb*-derived antigens and clonally expanded with the stimulation of the *Mtb*-derived antigens; this also demonstrated that the TRGV and TRGJ rearrangement is nonrandom and the diversity of the TCR γ genes in the functional γδ T cell repertoire may consist of specific TRGV-TRGJ combinations^48^.

Rearrangement of D genes is an important factor for the diversity of αβ T cells and γδ T cells repertoire^49^. Liu *et al*. had found that D-D fusion involved in TCRβ somatic recombination preferentially produced longer CDR3β loops and the D-D fusion was a key mechanism that contributed to longer TRB CDR3 loops^50^. However, in our study, we found that no differences in D-D fusion pattern existed in the three groups, and the frequency of D-D fusion was very low in most of the subjects, indicating that D-D fusion did not contribute to γδ TCR diversity in *Mtb* stimulation. This is in agreement with the perspective of Garboczi, who determined that although γδ T cells may have a large potential diversity of their repertoire and that D gene segments were also important for diversity, γδ T cells may not make the best use of that diversity^51^. This was also consistent with the length distribution of the TRD repertoire, which had no differences among the three groups.

In this study, we identified new preponderant CDR3 sequences in the LTBI group, the TB group, and the LTBI&TB group. The preponderant CDR3γ sequences in the TB group all used a Vγ9-JγP rearrangement. These results further suggested that γδ T cells expressing specific sequences whose CDR3γ used the Vγ9-JγP rearrangement may play an important role in *Mtb* infection and tuberculosis development. The little preponderant CDR3δ sequences in different groups may be caused by the greater diversity of the TRD repertoire. Due to the improved sequencing depth and stringent inclusion criteria, the preponderant CDR3 sequences we identified are expected to be more representative and more common. The dominant sequence we obtained with PCR in our previous study also appeared in this study, but it was not identified as a preponderant CDR3 sequence due to its relatively low frequency. These demonstrate that the traditional method we used in our previous study was reliable, and studying the γδ TCR repertoire with NGS is more advantageous. However, limited by sample resources, the number of subjects we recruited in this study was relatively less. The preponderant CDR3 sequences we identified need to be further validated with more subjects in the future.

Bukowski *et al*. had demonstrated that the junctional diversity (N and J region) of TCR γ chain was important for γδ TCR to recognize the antigens, and the functional γδ TCR repertoire partly depended upon preferentially rearranged Vγ-Jγ gene combinations^46^. According to our results, the frequency of γδ TCR with the rearranged Vγ9-JγP gene combinations increased obviously in the TB group, which suggested that the Vγ9-JγP gene combinations might be important for γδ T cells to recognize the antigens derived from mycobacteria. Furthermore, the marked γδ T cell expansion in response to mycobacteria stimulation was observed in both adult and newborn subjects, the TCR δ chain of the γδ T cells stimulated with mycobacteria had extensive junctional diversity, which meant the major mechanism of γδ T cell reactivity involves antigen recognition might be mediated by the germline-encoded segments of the TCR^52^. This might be the reason of the increasing diversity of the TRD CDR3 in TB group, and the increasing diversity of the TCR δ chain caused by the stimulation with *Mtb*-derived antigens might play important roles in immune response against TB infection. The changes of TCR γ chain and δ chain upon TB infection were also reflected by the preponderant sequences we obtained in TB group. With these preponderant sequences, we can screen more mycobacterial vaccine candidates for adjuvant through the reverse genetic strategy we have stablished in our previous study^17^, which could induce immune responses against TB infection.

In summary, we characterized the profile of the γδ TCR repertoire in patients with pulmonary tuberculosis at the level of sequence resolution for the first time with NGS. We found that the more increased the diversity of TRD, the longer CDR3 of TRG in the TB group, which may be caused by the shortened J trimming. We also found that the γδ T cells bearing Vγ9-JγP rearrangement clonally expanded, and the γ chain of the γδ TCR may be more important in antigen recognition than the δ chain. The results promote our understanding of the profile of the γδ TCR repertoire in TB patients. Taking advantage of the analytical power of NGS, this study gave us a comprehensive understanding of the γδ T cells repertoire changes during *Mtb* infection, and may provide useful information about the γδ T cell-mediated immune response against *Mtb*. Moreover, we found some preponderant sequences in TB patients, which may also shed light on the development of new vaccines and adjuvants against TB.

## Materials and Methods

### Ethics statement

This work received approval from the Clinical Ethics Committee of the Institute of Pathogen Biology, the Chinese Academy of Medical Sciences, and Beijing Union Medical College. Written informed consent was obtained from all participants. All of the subjects gave written informed consent in accordance with the Declaration of Helsinki.

### Study groups

Three study groups (HC: healthy control; LTBI: latent tuberculosis patients; and TB: active pulmonary tuberculosis patients) were enlisted to investigate the detailed characteristics of the γδ TCR repertoire. The general information and clinical characteristics of the recruited participants are summarized in Table 1.

The participants in the TB group were all in the acute phase; they were newly diagnosed pulmonary tuberculosis patients without prior anti-tuberculosis treatment and with the following clinical parameters: presence of cough/expectoration, a chest x-ray showing infiltration and/or cavities, a minimum of one positive sputum smear, and a positive culture result for acid-fast bacilli, and/or enzyme-linked immunospot assay (ELISpot) detecting IFN-γ release by QuantiFERON-TB Gold kit (QIAGEN, Germantown, USA). This kit measures the cell-mediated response to highly specific TB antigens (ESAT-6/CFP-10/TB-7.7 (p4)) that are associated with M. tuberculosis infection and proven unaffected by the prior BCG vaccination and most non-TB mycobacteria. Participants in the LTBI group were members of the medical staff of the Sixth People’s Hospital of Zhengzhou. They were asymptomatic and had T-cell responses specific to IFN-γ detected by ELISpot. The HC group consisted of healthy volunteer subjects who did not have any changes noted on x-ray and did not have a history of tuberculosis or any other underlying disease. The subjects with viral and other bacterial infections/co-infections, severe hepatic diseases, and renal diseases, especially immunological or autoimmune diseases, were excluded. Diabetes mellitus, medication, pregnancy, and smoking were also included in the exclusion criteria.

### Sample preparation

#### Isolating γδ T Cells

Peripheral blood mononuclear cells (PBMCs) were isolated from peripheral blood by density gradient cell separation using Human Lymphocyte Separation tubes (Dakewei Biotech, Shenzhen, China) according to the manufacturer’s instructions. Then, the γδ T cells were isolated from the PBMCs with the Anti-TCRγ/δ MicroBead Kit (Miltenyi Biotec, Teterow, Germany) as follows: the PBMCs were incubated with Anti-TCR γ/δ Hapten-Antibody, followed by fluorescence and magnetic labeling with Anti-Hapten Micro-Beads-FITC. The cell suspension incubated with antibody was then loaded on a column, which was placed in the magnetic field. The magnetically labeled γδ T cells were retained and the unlabeled cells run through. After removal of the column from the magnetic field, the magnetically retained γ/δ^+^ cells were then eluted and immediately resuspended in Trizol reagent (Life Technologies, Carlsbad, CA, USA). Before sample collection, the efficiency of the magnetic bead-based purification of gamma delta T cells was tested by flow cytometric analysis and the results showed that the purity of isolated γδ T cells was about 97%. During the sample collection and isolation, the isolated γδ T cells from each sample were counted and the ratio of γδ T cells/PBMC in each sample was calculated. The ratio range was from 1.3% to 4.9% and all of them were within the reasonable scope of 0.5–10%.

#### Reverse transcription PCR (RT-PCR) and sequencing

RT-PCR and sequencing were done by iRepertoire (Huntsville, AL, USA). Specifically, total RNA of the γδ T cells was extracted with Trizol reagent and subjected to RT-PCR using a One-step RT-PCR kit (Qiagen Inc., Hilden, Germany). cDNA was then subjected to Amplicon rescued multiplex PCR (ARM-PCR) using human T cell gamma receptor primers and human T cell delta receptor primers according to the manufacturer’s instructions (iRepertoire Inc., Huntsville, AL, USA). Information about the primers can be found in the United States Patent and Trademark Office (US9012148B2). The PCR products were run on 2% agarose gel and the DNA at 212–280 bp was extracted from the gel using a Qiagen Gel Extraction kit. After assessing the DNA samples, 10 sample libraries were pooled and sequenced using the Illumina MiSeq platform (Illumina, San Diego, CA, USA).

### Analysis of sequencing data

The raw data were analyzed by iRepertoire using IRmap programs with a modified Smith-Waterman algorithm. The CDR3 was identified as the interval between two conserved amino acids for the TCR gamma and delta chain, respectively. The best matches of the germline V gene and J gene and the information of the V trimming and J trimming, and the addition of nontemplated nucleotides (N addition) were searched by determining alignments between the sequencing products and germline sequences in the IMGT/GENE-DB databases as previously described^53^. iRepertoire® provided filtered DNA sequences. The length of the CDR3 region, information on the V, D, and J gene segment usage, D-D fusion pattern of TRD, and the length of the V trimming, J trimming and N addition, were obtained from these filtered sequences. The total CDR3 sequences referred to all of the CDR3 sequences obtained from the filtered sequences and the unique CDR3 sequences referred to each distinct CDR3 sequence regardless of how many copies appeared.

### Statistical analyses

The Mann–Whitney test was used to assess differences between any two of the three groups. Analysis was performed with PRISM version 6 (Graph Pad) and Origin Pro. Significant differences among each fraction in all of the figures are indicated by asterisks: significant, *0.01 ≤ *p* < 0.05; very significant, **p < 0.01.

## Electronic supplementary material

Supplementary materials

## References

[1] WHO, *Global Tuberculosis Report*. (Switzerland: WHO, World Health Organization, 2016).

[2] Rao M (2017). Mycobacterium tuberculosis proteins involved in cell wall lipid biosynthesis improve BCG vaccine efficacy in a murine TB model. Int J Infect Dis.

[3] Moliva JI, Turner J, Torrelles JB (2015). Prospects in Mycobacterium bovis Bacille Calmette et Guerin (BCG) vaccine diversity and delivery: why does BCG fail to protect against tuberculosis?. Vaccine.

[4] Prezzemolo T (2014). Functional Signatures of Human CD4 and CD8 T Cell Responses to Mycobacterium tuberculosis. Front Immunol.

[5] Meraviglia S, El DS, Dieli F, Martini F, Martino A (2011). gammadelta T cells cross-link innate and adaptive immunity in Mycobacterium tuberculosis infection. Clin Dev Immunol.

[6] Chen ZW (2013). Multifunctional immune responses of HMBPP-specific Vgamma2Vdelta2 T cells in M. tuberculosis and other infections. Cell Mol Immunol.

[7] Janis EM, Kaufmann SH, Schwartz RH, Pardoll DM (1989). Activation of gamma delta T cells in the primary immune response to Mycobacterium tuberculosis. Science.

[8] Casetti R, Martino A (2008). The plasticity of gamma delta T cells: innate immunity, antigen presentation and new immunotherapy. Cell Mol Immunol.

[9] Holtmeier W, Kabelitz D (2005). gammadelta T cells link innate and adaptive immune responses. Chem Immunol Allergy.

[10] Davey MS (2011). Human neutrophil clearance of bacterial pathogens triggers anti-microbial gammadelta T cell responses in early infection. Plos Pathog.

[11] Nunes-Alves C (2014). In search of a new paradigm for protective immunity to TB. Nat Rev Microbiol.

[12] Yuk JM, Jo EK (2014). Host immune responses to mycobacterial antigens and their implications for the development of a vaccine to control tuberculosis. Clin Exp Vaccine Res.

[13] Shen Y (2002). Adaptive immune response of Vgamma2Vdelta2+ T cells during mycobacterial infections. Science.

[14] Ferreira LM (2013). Gammadelta T cells: innately adaptive immune cells?. Int Rev Immunol.

[15] Spencer CT, Abate G, Blazevic A, Hoft DF (2008). Only a subset of phosphoantigen-responsive gamma9delta2 T cells mediate protective tuberculosis immunity. J Immunol.

[16] Xi X, Han X, Li L, Zhao Z (2011). gammadelta T cells response to Mycobacterium tuberculosis in pulmonary tuberculosis patients using preponderant complementary determinant region 3 sequence. Indian J Med Res.

[17] Xi X (2011). A novel strategy to screen Bacillus Calmette-Guerin protein antigen recognized by gammadelta TCR. Plos One.

[18] Xi X, Han X, Li L, Zhao Z (2013). Identification of a new tuberculosis antigen recognized by gammadelta T cell receptor. Clin Vaccine Immunol.

[19] Six A (2013). The past, present, and future of immune repertoire biology - the rise of next-generation repertoire analysis. Front Immunol.

[20] Chaudhry S, Cairo C, Venturi V, Pauza CD (2013). The gammadelta T-cell receptor repertoire is reconstituted in HIV patients after prolonged antiretroviral therapy. Aids.

[21] Di Sante G (2015). Collagen Specific T-Cell Repertoire and HLA-DR Alleles: Biomarkers of Active Refractory Rheumatoid Arthritis. EBioMedicine.

[22] O’Connell AE (2014). Next generation sequencing reveals skewing of the T and B cell receptor repertoires in patients with wiskott-Aldrich syndrome. Front Immunol.

[23] Schrama, D., Ritter, C. & Becker, J. C. T cell receptor repertoire usage in cancer as a surrogate marker for immune responses. *Semin Immunopathol* (2017). 10.1007/s00281-016-0614-9 28074285

[24] Li B (1996). Disease-specific changes in gammadelta T cell repertoire and function in patients with pulmonary tuberculosis. J Immunol.

[25] Dieli F (1999). Predominance of Vgamma9/Vdelta2 T lymphocytes in the cerebrospinal fluid of children with tuberculous meningitis: reversal after chemotherapy. Mol Med.

[26] Krell PF (2013). Next-generation-sequencing-spectratyping reveals public T-cell receptor repertoires in pediatric very severe aplastic anemia and identifies a beta chain CDR3 sequence associated with hepatitis-induced pathogenesis. Haematologica.

[27] Shmigol AV, Eisner DA, Wray S (1999). The role of the sarcoplasmic reticulum as a Ca2+ sink in rat uterine smooth muscle cells. J Physiol.

[28] Yang J (2013). Limited T cell receptor beta variable repertoire responses to ESAT-6 and CFP-10 in subjects infected with Mycobacterium tuberculosis. Tuberculosis (Edinb).

[29] Adams EJ, Gu S, Luoma AM (2015). Human gamma delta T cells: Evolution and ligand recognition. Cell Immunol.

[30] Balbi B (1993). T-lymphocytes with gamma delta+ V delta 2+ antigen receptors are present in increased proportions in a fraction of patients with tuberculosis or with sarcoidosis. Am Rev Respir Dis.

[31] Chen ZW, Letvin NL (2003). Adaptive immune response of Vgamma2Vdelta2 T cells: a new paradigm. Trends Immunol.

[32] Chen ZW (2016). Protective immune responses of major Vgamma2Vdelta2 T-cell subset in M. tuberculosis infection. Curr Opin Immunol.

[33] Esin S (1996). Proliferation of distinct human T cell subsets in response to live, killed or soluble extracts of Mycobacterium tuberculosis and Myco. avium. Clin Exp Immunol.

[34] Huang D (2012). Clonal immune responses of Mycobacterium-specific gammadelta T cells in tuberculous and non-tuberculous tissues during M. tuberculosis infection. Plos One.

[35] Miconnet I (2012). Probing the T-cell receptor repertoire with deep sequencing. Curr Opin Hiv Aids.

[36] Boyd, S. D. & Joshi, S. A. High-Throughput DNA Sequencing Analysis of Antibody Repertoires. *Microbiol Spectr* **2** (2014). 10.1128/microbiolspec.AID-0017-2014 26104353

[37] Hou XL, Wang L, Ding YL, Xie Q, Diao HY (2016). Current status and recent advances of next generation sequencing techniques in immunological repertoire. Genes Immun.

[38] Li D (2014). Profiling the T-cell receptor repertoire of patient with pleural tuberculosis by high-throughput sequencing. Immunol Lett.

[39] Rock EP, Sibbald PR, Davis MM, Chien YH (1994). CDR3 length in antigen-specific immune receptors. J Exp Med.

[40] Hayday AC (2000). [gamma][delta] cells: a right time and a right place for a conserved third way of protection. Annu Rev Immunol.

[41] Adams EJ, Chien YH, Garcia KC (2005). Structure of a gammadelta T cell receptor in complex with the nonclassical MHC T22. Science.

[42] Xu B (2011). Crystal structure of a gammadelta T-cell receptor specific for the human MHC class I homolog MICA. Proc Natl Acad Sci USA.

[43] Uldrich AP (2013). CD1d-lipid antigen recognition by the gammadelta TCR. Nat Immunol.

[44] Xu C (2007). Gammadelta T cells recognize tumor cells via CDR3delta region. Mol Immunol.

[45] Kabelitz D (1991). The primary response of human gamma/delta+ T cells to Mycobacterium tuberculosis is restricted to V gamma 9-bearing cells. J Exp Med.

[46] Bukowski JF, Morita CT, Band H, Brenner MB (1998). Crucial role of TCR gamma chain junctional region in prenyl pyrophosphate antigen recognition by gamma delta T cells. J Immunol.

[47] Van Rhijn I (2007). Highly diverse TCR delta chain repertoire in bovine tissues due to the use of up to four D segments per delta chain. Mol Immunol.

[48] Kohsaka H, Chen PP, Taniguchi A, Ollier WE, Carson DA (1993). Regulation of the mature human T cell receptor gamma repertoire by biased V-J gene rearrangement. J Clin Invest.

[49] Allison JP, Havran WL (1991). The immunobiology of T cells with invariant gamma delta antigen receptors. Annu Rev Immunol.

[50] Liu P (2014). Characterization of human alphabetaTCR repertoire and discovery of D-D fusion in TCRbeta chains. Protein Cell.

[51] Garboczi DN (2005). Structural biology. “D” is not for diversity. Science.

[52] Panchamoorthy G (1991). A predominance of the T cell receptor V gamma 2/V delta 2 subset in human mycobacteria-responsive T cells suggests germline gene encoded recognition. J Immunol.

[53] Wang C (2010). High throughput sequencing reveals a complex pattern of dynamic interrelationships among human T cell subsets. Proc Natl Acad Sci USA.

