# Author Correction: Next generation sequencing reveals changes of the γδ T cell receptor repertoires in patients with pulmonary tuberculosis

**DOI:** 10.1038/s41598-018-24593-8

**Published:** 2018-04-16

**Authors:** Chaofei Cheng, Bei Wang, Lei Gao, Jianmin Liu, Xinchun Chen, He Huang, Zhendong Zhao

**Affiliations:** 1 MOH Key Laboratory of Systems Biology of Pathogens, Institute of Pathogen Biology, and Centre for Tuberculosis Research, Chinese Academy of Medical Sciences & Peking Union Medical College, Beijing, 100730 China; 2 Clinical Immunology Center, Chinese Academy of Medical Sciences & Peking Union Medical College, Beijing, 100730 China; 3 CAMS-Oxford University International Center for Translational Immunology, Chinese Academy of Medical Sciences & Peking Union Medical College, Beijing, 100730 China; 4 The Sixth People’s Hospital of Zhengzhou, Zhengzhou, 450015 China; 5 Department of Pathogen Biology, School of Medicine, Shenzhen University, Shenzhen, 518002 China

Correction to: *Scientific Reports* 10.1038/s41598-018-22061-x, published online 02 March 2018

The Acknowledgements section in this Article is incomplete.

“This work was supported by the CAMS Innovation Fund for Medical Sciences [CIFMS 2016-12M-1-013], the National Natural Science Foundation of China [No. 31379144], and the Twelfth-Fifth Mega-Scientific Project on the prevention and treatment of AIDS, viral hepatitis, and other infectious diseases in China [No. 2012ZX10003002].”

should read:

“This work was supported by the CAMS Innovation Fund for Medical Sciences [CIFMS 2016-12M-1-013 and 2016-12M-1-014], the National Natural Science Foundation of China [No.31370890], and the Twelfth-Fifth Mega-Scientific Project on the prevention and treatment of AIDS, viral hepatitis, and other infectious diseases in China [No. 2012ZX10003002].”

