# Supplementary material for: Next generation sequencing reveals changes of the γδ T cell receptor repertoires in patients with pulmonary tuberculosis
Source: Sci Rep. 2018 Mar 2;8:3956. doi: 10.1038/s41598-018-22061-x (PMC5834497; doi:10.1038/s41598-018-22061-x)
Supplement: Supplementary file 1 — Supplementary materials [file 41598_2018_22061_MOESM1_ESM.pdf]

# *Supplementary Material*

# Next generation sequencing reveals changes of the γδ T cell receptor repertoires in patients with pulmonary tuberculosis

**Chaofei Cheng, Bei Wang, Lei Gao, Jianmin Liu， Xinchun Chen, He Huang, Zhendong Zhao**

## 1 Supplementary Figures

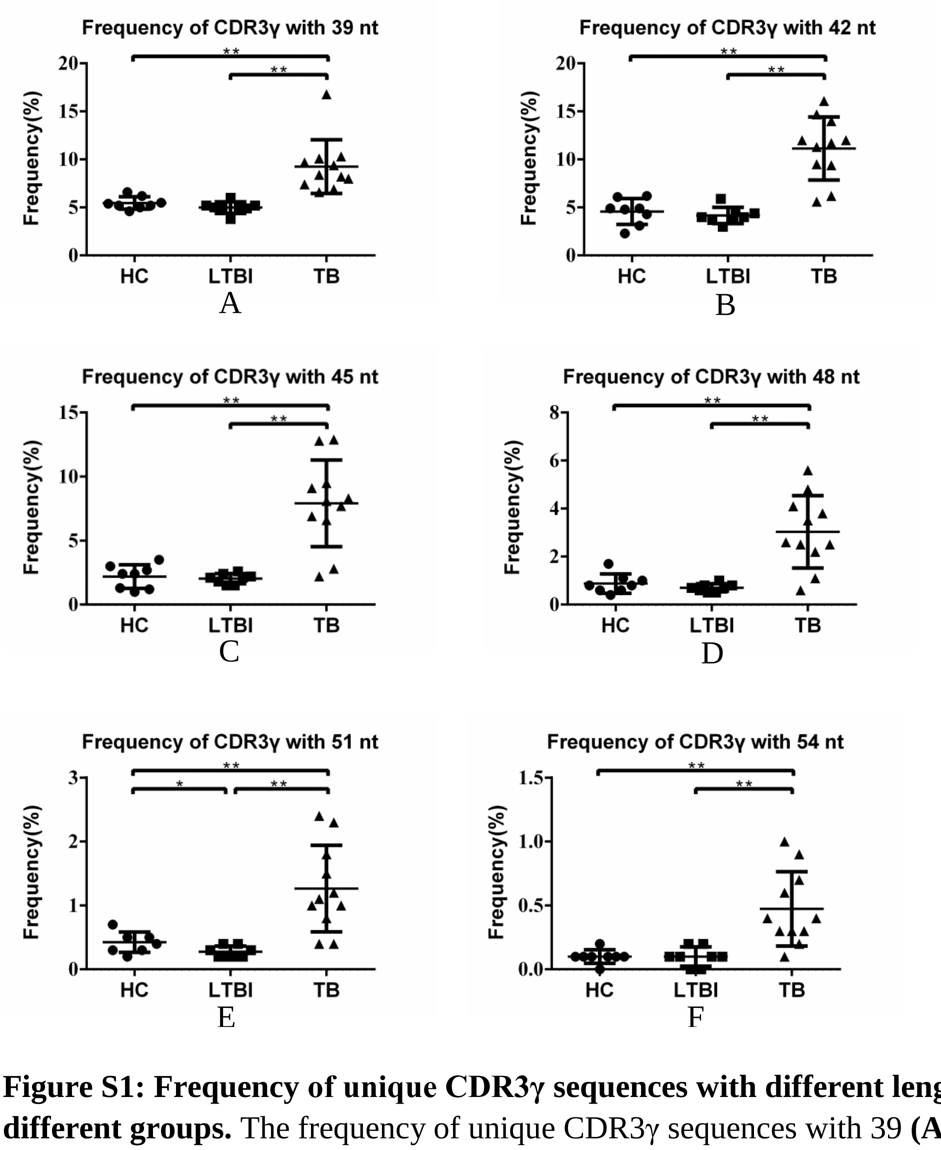


**Figure S1: Frequency of unique CDR3γ sequences with different lengths in different groups.** The frequency of unique CDR3γ sequences with 39 **(A)**, 42 **(B)**, 45 **(C)**, 48 **(D)**, 51 **(E)**, and 54 **(F)** nucleotides in different groups.

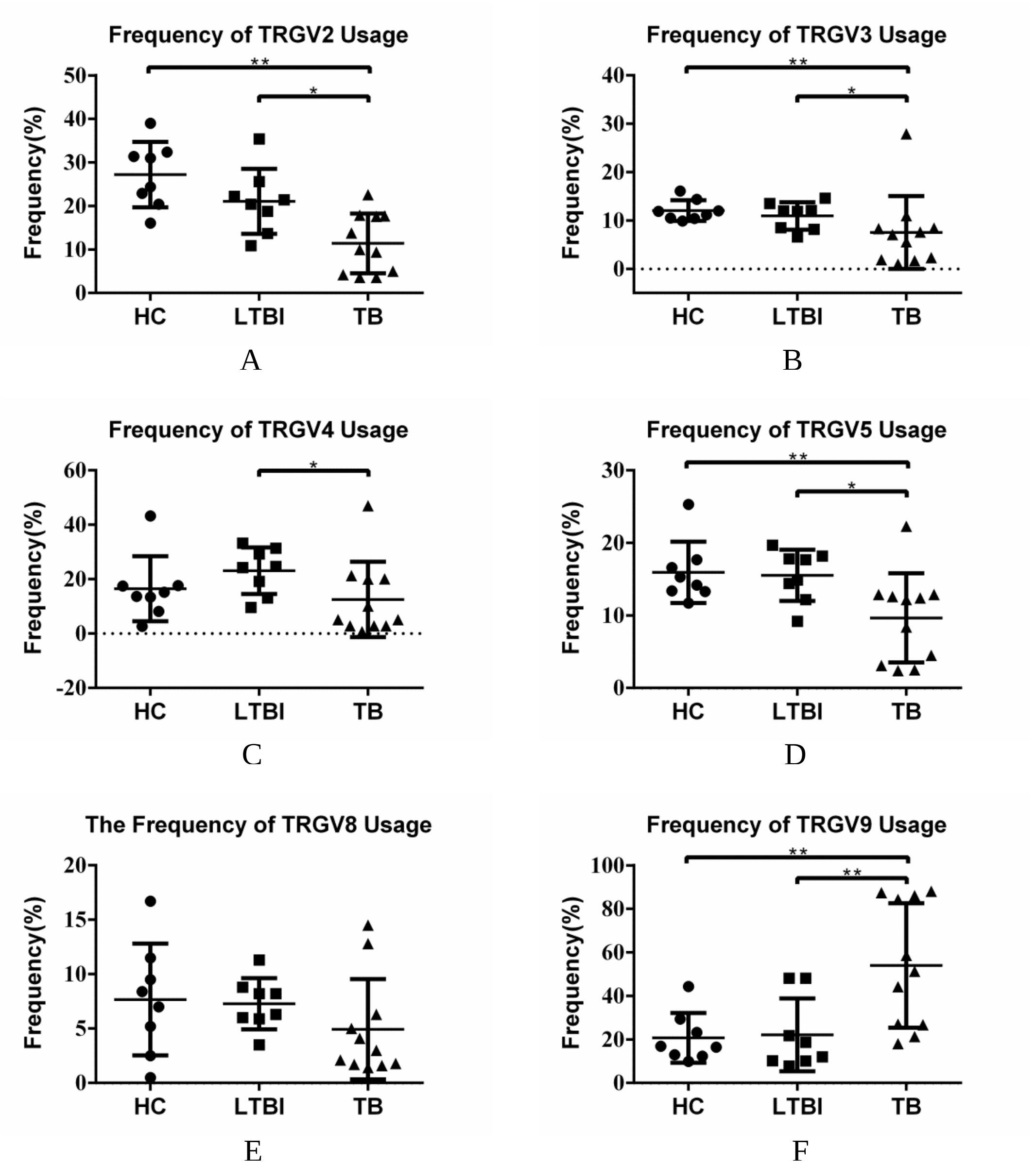


**Figure S2: Usage of TRGV genes in total CDR3 sequences in different groups.** Frequency of usage of TRGV2 **(A)**, TRGV3 **(B)**, TRGV4 **(C)**, TRGV5 **(D)**, TRGV8 **(E)**, and TRGV9 **(F)** in total CDR3 sequences in different groups.

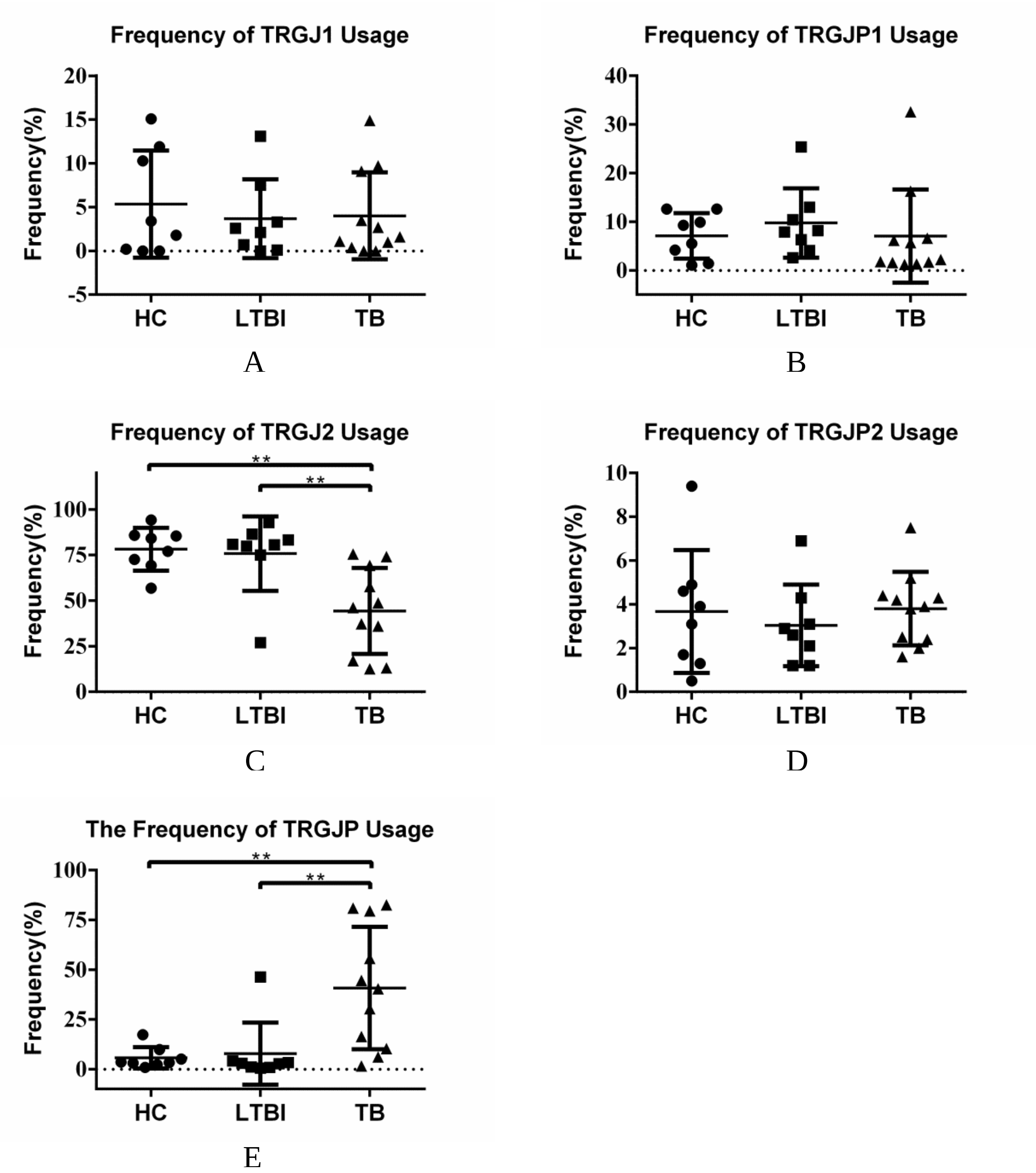


**Figure S3: Usage of TRGJ genes in total CDR3 sequences in different groups**. Frequency of usage of TRGJ1 **(A)**, TRGJP1 **(B)**, TRGJ2 **(C)**, TRGJP2 **(D),** and TRGJP **(E)** in total CDR3 sequences from different groups.

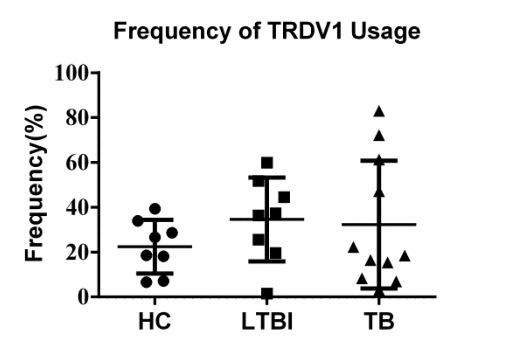


A

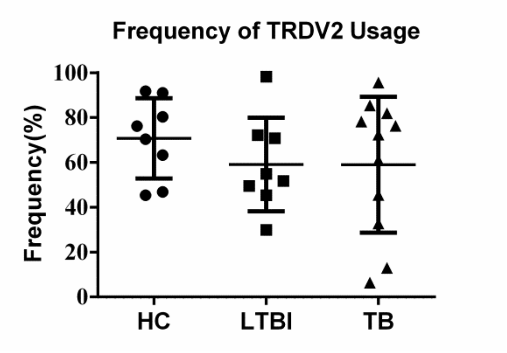


B

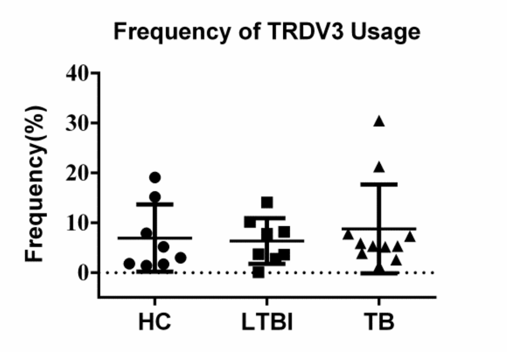


C

**Figure S4: Usage of TRDV genes in total CDR3 sequences in different groups.** Frequency of usage of TRDV1 **(A)**, TRDV2 **(B)**, and TRDV3 **(C)** in total CDR3 sequences in different groups.

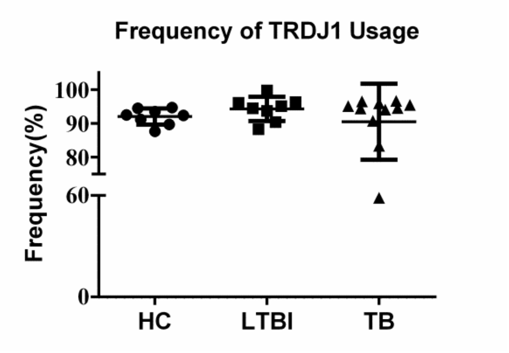


A

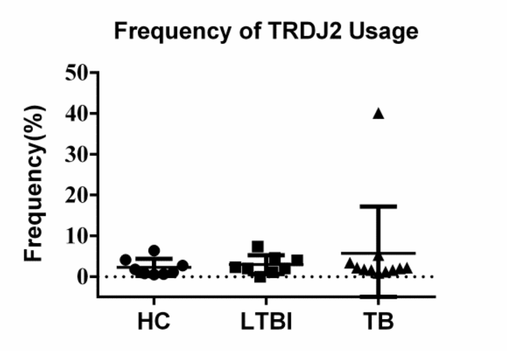


B

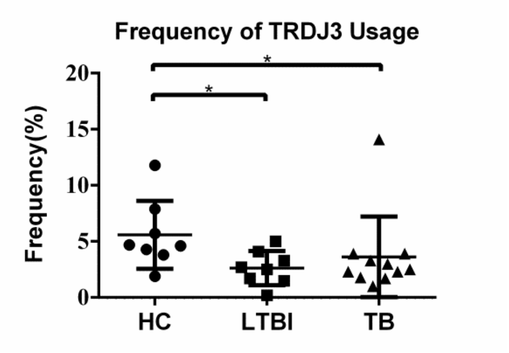


C

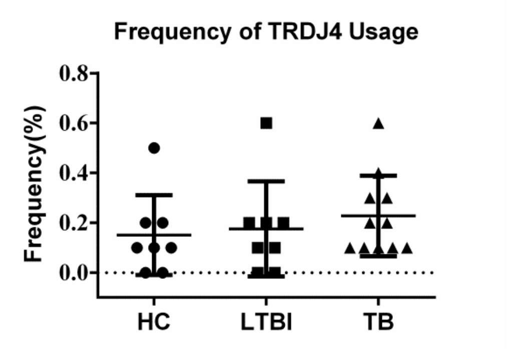


D

**Figure S5: Usage of TRDJ genes in total CDR3 sequences in different groups.** Frequency of usage of TRDJ1 **(A)**, TRDJ2 **(B)**, TRDJ3 **(C)**, and TRDJ4 **(D)** in total CDR3 sequences in different groups.

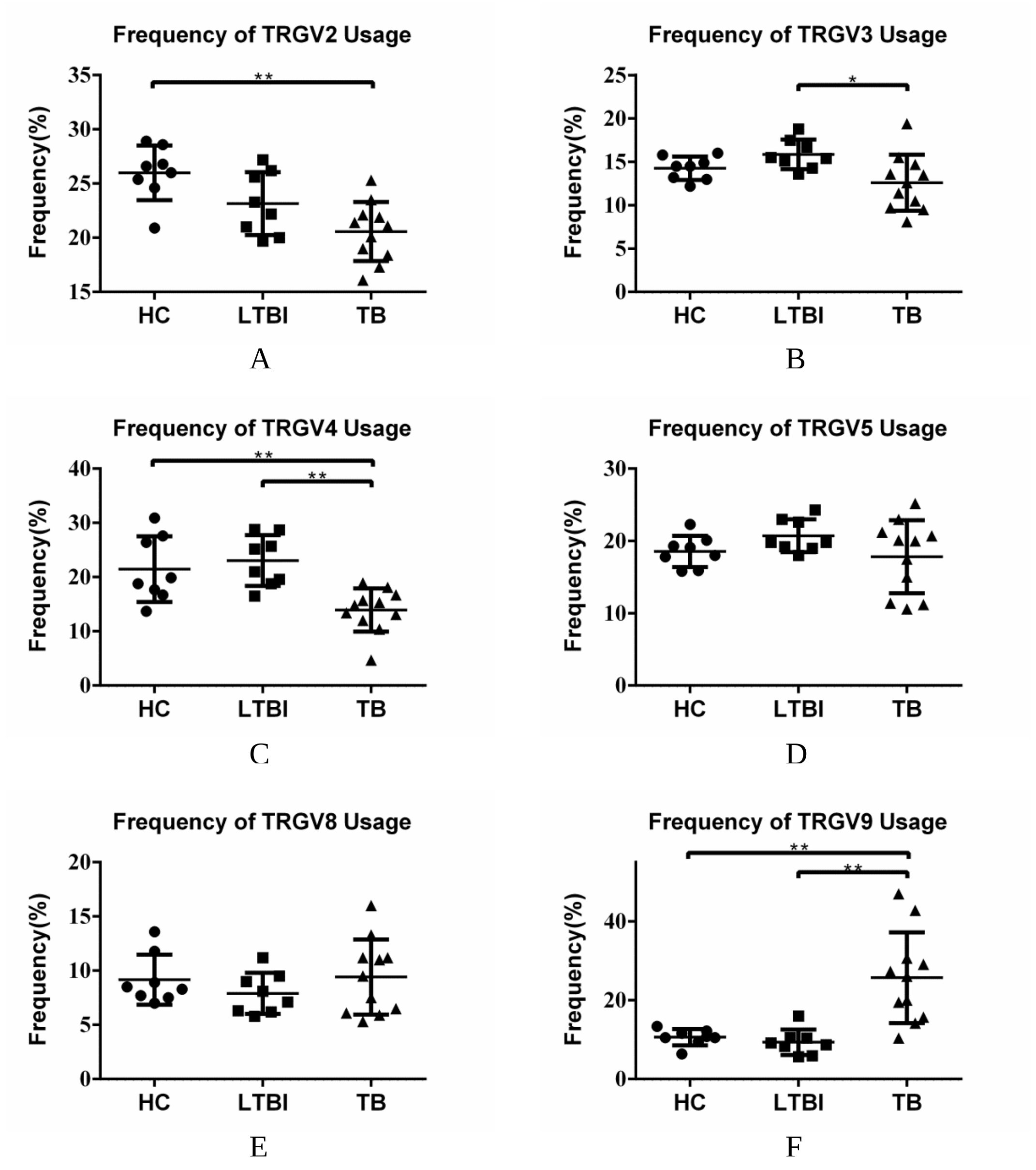


**Figure S6: Usage of TRGV genes in unique CDR3 sequences in different groups.** Frequency of usage of TRGV2 **(A)**, TRGV3 **(B)**, TRGV4 **(C)**, TRGV5 **(D)**, TRGV8 **(E)**, and TRGV9 **(F)** in unique CDR3 sequences in different groups.

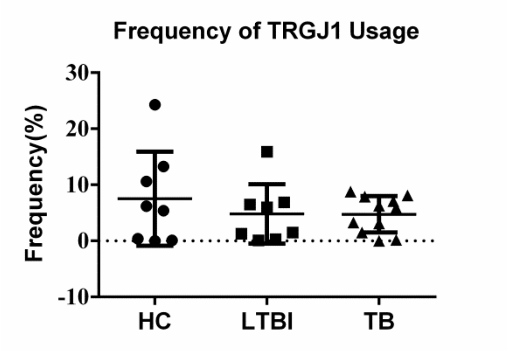


A

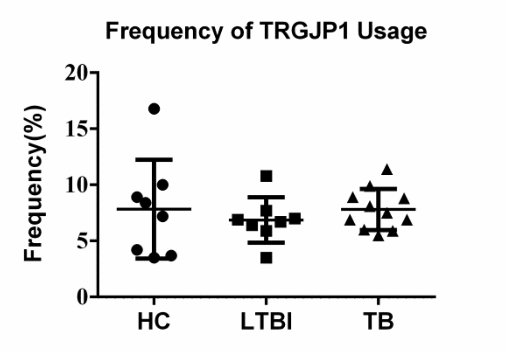


B

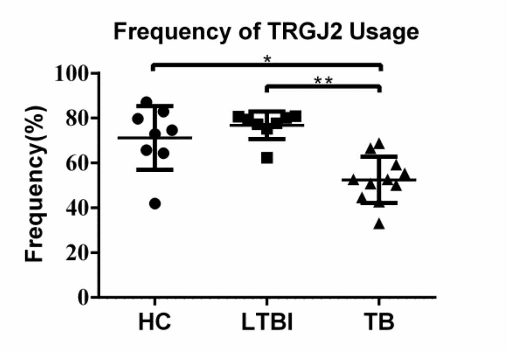


C

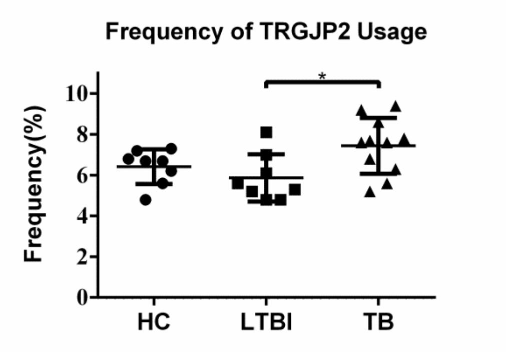


D

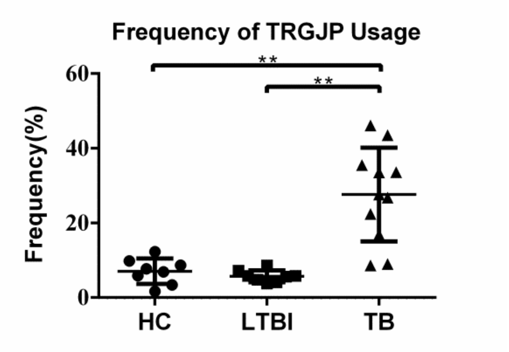


E

**Figure S7: Usage of TRGJ genes in unique CDR3 sequences in different groups.** Frequency of usage of TRGJ1 **(A)**, TRGJP1 **(B)**, TRGJ2 **(C)**, TRGJP2 **(D)**, and TRGJP **(E)** in unique CDR3 sequences in different groups.

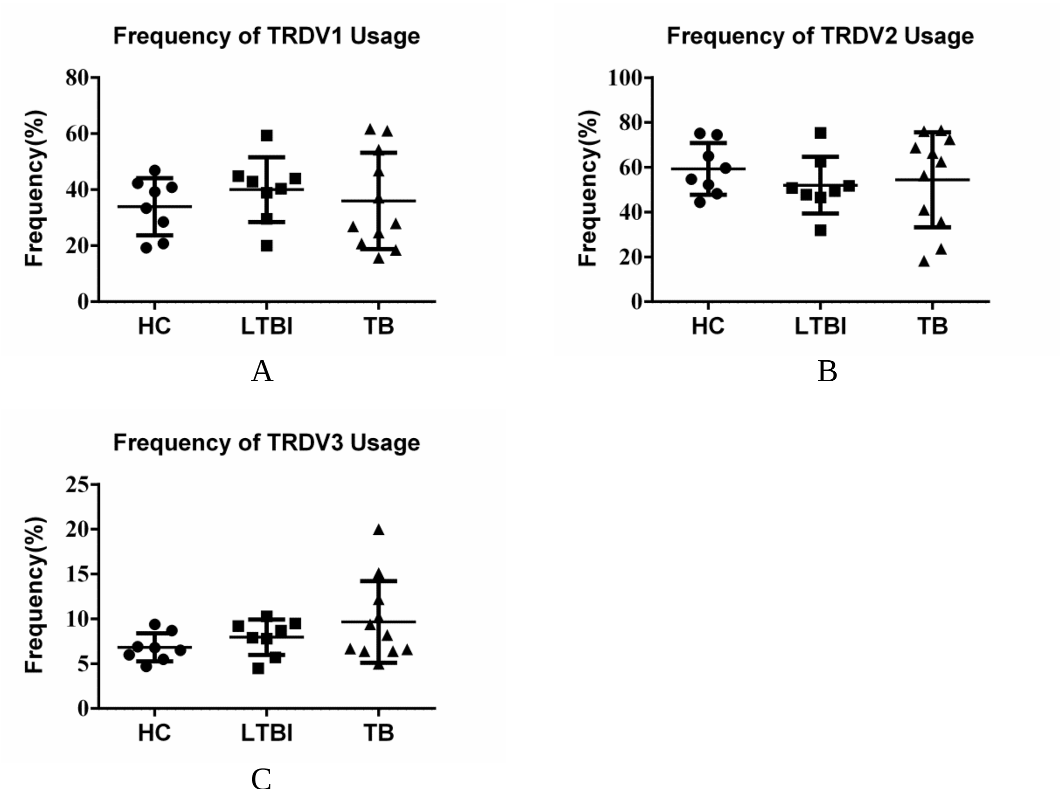


**Figure S8: Usage of TRDV genes in unique CDR3 sequences in different groups.** Frequency of usage of TRDV1 **(A)**, TRDV2 **(B)**, and TRDV3 **(C)** in unique CDR3 sequences in different groups.

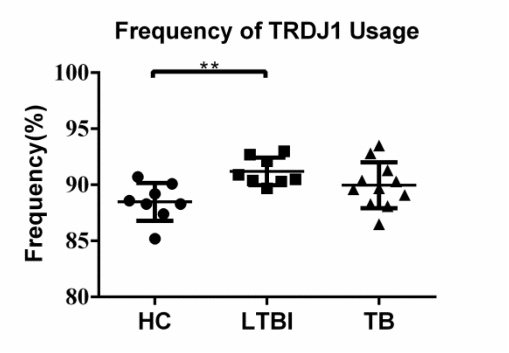


A

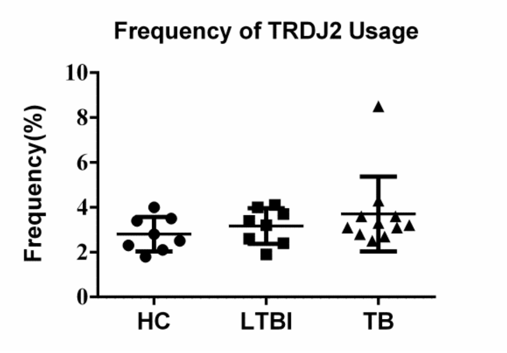


B

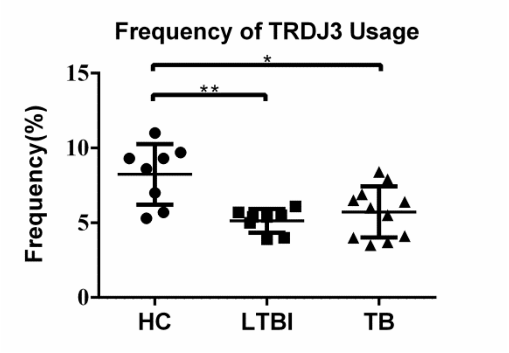


C

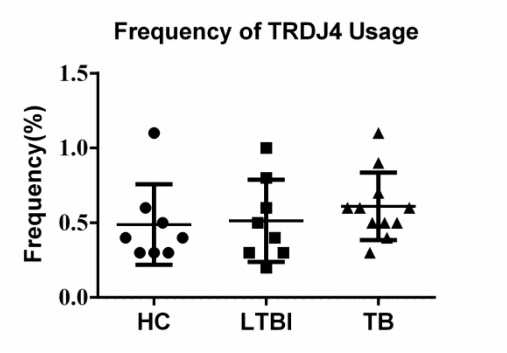


D

**Figure S9: Usage of TRDJ genes in unique CDR3 sequences in different groups.** Frequency of usage of TRDJ1 **(A)**, TRDJ2 **(B)**, TRDJ3 **(C)**, and TRDJ4 **(D)** in unique CDR3 sequences in different groups.

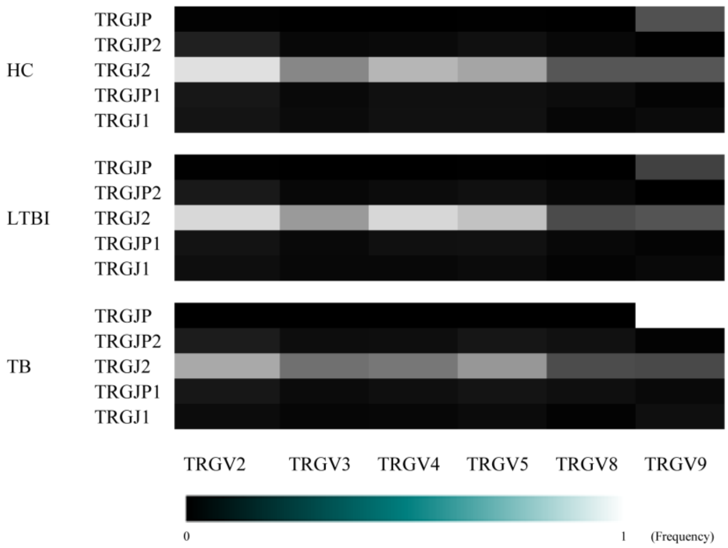


A

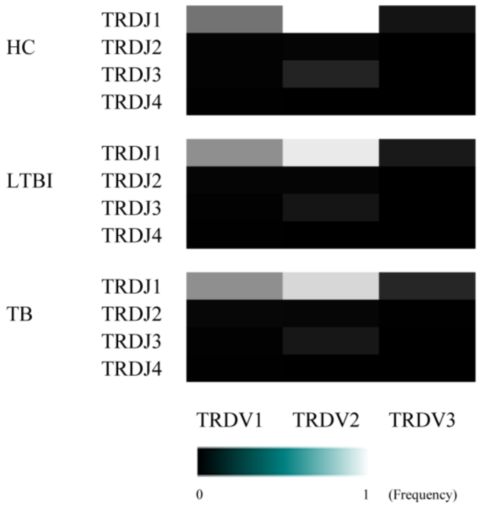


B

**Figure S10: Analysis of the combinatorial diversity of TRG and TRD in unique CDR3 sequences in different groups. (A)** Frequencies of specific TRGV and TRGJ gene paring in different groups. **(B)** Frequencies of specific TRDV and TRDJ gene paring in different groups.

## 2 Supplementary table

### Table S1 Detailed information of reads, CDR3 reads, and Unique CDR3 reads for TRG (A) and TRD (B)

A

| Healthy control (HC) | | | | Latent tuberculosis infection (LTBI) | | | | Active Tuberculosis (TB) | | | |
|---|---|---|---|---|---|---|---|---|---|---|---|
| Sample ID | TRG | | | Sample ID | TRG | | | Sample ID | TRG | | |
| | Reads | CDR3 | Unique CDR3 | | Reads | CDR3 | Unique CDR3 | | Reads | CDR3 | Unique CDR3 |
| 101 | 527,145 | 393,670 | 2,402 | 206 | 433,684 | 312,374 | 2,340 | 106 | 534,133 | 488,209 | 3,877 |
| 102 | 400,332 | 207,860 | 2,697 | 207 | 418,113 | 241,297 | 2,285 | 107 | 506,186 | 248,519 | 3,450 |
| 103 | 518,460 | 216,253 | 2,707 | 208 | 424,662 | 248,089 | 2,369 | 108 | 474,739 | 437,458 | 6,274 |
| 104 | 441,260 | 212,202 | 2,338 | 209 | 390,266 | 168,703 | 4,913 | 109 | 515,852 | 493,804 | 4,283 |
| 105 | 396,793 | 204,893 | 1,988 | 210 | 494,214 | 380,029 | 2,733 | 110 | 511,539 | 457,248 | 3,067 |
| 201 | 447,892 | 388,826 | 905 | 308 | 583,553 | 454,033 | 2,022 | 301 | 439,880 | 298,600 | 757 |
| 202 | 385,373 | 254,602 | 5,956 | 309 | 486,936 | 233,988 | 5,209 | 302 | 518,576 | 462,688 | 2,538 |
| 203 | 434,758 | 274,741 | 4,190 | 310 | 531,258 | 173,200 | 2,482 | 303 | 443,181 | 227,075 | 2,636 |
| 204 | 412,789 | 99,642 | 904 | | | | | 304 | 439,424 | 386,280 | 3,282 |
| 205 | 412,819 | 175,324 | 3,828 | | | | | 305 | 521,340 | 479,223 | 3,871 |
| | | | | | | | | 306 | 469,363 | 383,927 | 8,636 |
| | | | | | | | | 307 | 557,217 | 470,500 | 3,298 |

B

| Healthy control (HC) | | | | Latent tuberculosis infection (LTBI) | | | | Active Tuberculosis (TB) | | | |
|---|---|---|---|---|---|---|---|---|---|---|---|
| Sample ID | TRD | | | Sample ID | TRD | | | Sample ID | TRD | | |
| | Reads | CDR3 | Unique CDR3 | | Reads | CDR3 | Unique CDR3 | | Reads | CDR3 | Unique CDR3 |
| 101 | 532,038 | 510,820 | 4,049 | 206 | 316,683 | 304,219 | 3,827 | 106 | 348,499 | 335,216 | 8,314 |
| 102 | 492,231 | 481,687 | 4,103 | 207 | 386,984 | 367,631 | 6,896 | 107 | 343,415 | 331,990 | 6,776 |
| 103 | 467,490 | 449,545 | 4,145 | 208 | 244,783 | 229,890 | 2,437 | 108 | 405,106 | 396,472 | 18,248 |
| *104 | 101,641 | 98,274 | 1,240 | 209 | 239,671 | 224,289 | 4,160 | 109 | 302,032 | 296,017 | 8,556 |
| 105 | 358,211 | 336,421 | 5,276 | 210 | 283,100 | 270,454 | 3,568 | 110 | 366,972 | 363,263 | 4,219 |
| 201 | 407,928 | 388,536 | 1,849 | 308 | 607,579 | 606,000 | 1,196 | 301 | 326,086 | 319,531 | 2,474 |
| 202 | 422,724 | 407,607 | 5,552 | 309 | 121,656 | 108,243 | 2,263 | 302 | 485,011 | 472,377 | 6,552 |
| 203 | 397,725 | 387,306 | 4,527 | 310 | 373,848 | 355,101 | 1,310 | 303 | 388,316 | 365,575 | 5,511 |
| *204 | 232,16 | 21,654 | 868 | | | | | *304 | 1,510 | 1,437 | 323 |
| 205 | 327,426 | 312,635 | 2,681 | | | | | 305 | 389,007 | 372,789 | 10,645 |
| | | | | | | | | 306 | 464,525 | 453,672 | 24,064 |
| | | | | | | | | 307 | 461,553 | 452,617 | 8,913 |

*Data obtained from samples 104, 204, and 304 were disregarded.

## Table S2 Statistics of TRG and TRD sequences

| | | TRG | | | TRD | | |
|---|---|---|---|---|---|---|---|
| | | HC | LTBI | TB | HC | LTBI | TB |
| Reads | Total | 3,523,572 | 3,762,686 | 5,492,006 | 3,405,773 | 2,574,304 | 4,280,522 |
| | Mean | 440,447 ±54,774 | 470,336 ±65,403 | 499,273 ±37,518 | 425,722 ±68,453 | 321,788 ±142,872 | 389,138 ±60,142 |
| CDR3 | Total | 2,116,169 | 2,211,713 | 4,447,251 | 3,274,577 | 2,465,827 | 4,159,519 |
| | Mean | 264,521 ±83,995 | 276,464 ±99,763 | 404,296 ±99,812 | 409,320 ±68,389 | 308,228 ±145,903 | 378,138 ±59,184 |
| Unique CDR3 | Total | 24,673 | 24,353 | 42,687 | 32,182 | 25,657 | 104,272 |
| | mean | 3,084 ±1,546 | 3,044 ±1,263 | 3,881 ±2,069 | 4,023 ±1,237 | 3,207 ±1,858 | 9,479 ±6,339 |

**Table S3 One way Anova of the age of each subjects between different groups**

| | Sum of Squares | df | Mean Square | F | Sig. |
|---|---|---|---|---|---|
| Between Groups | 1346.938 | 2 | 673.469 | 6.321 | 0.006 |
| Within Groups | 2557.136 | 24 | 106.577 | | |
| Total | 3904.074 | 26 | | | |

**Table S4 Post Hoc Multiple Comparisons of the age age of each subject between different groups**

| Group | N | Subset for alpha=0.05 | |
|---|---|---|---|
| | | 1 | 2 |
| TB | 11 | 28.82 | |
| HC | 8 | 30.75 | |
| LTBI | 8 | | 45.00 |
| Sig. | | 0.698 | 1.000 |
